# Intercellular crosstalk between cancer cells and cancer-associated fibroblasts via extracellular vesicles

**DOI:** 10.1186/s12935-022-02784-8

**Published:** 2022-11-24

**Authors:** Yutaka Naito, Yusuke Yoshioka, Takahiro Ochiya

**Affiliations:** 1 Department of Bioregulation, Institute for Advanced Medical Sciences, Nippon Medical School, 1-1-5, Sendagi, Bunkyo-Ku, Tokyo, 113-8602 Japan; 2 Department of Molecular and Cellular Medicine, Institute of Medical Science, Tokyo Medical University, 6-7-1, Nishishinjuku, Shinjuku-Ku, Tokyo, 160-0023 Japan

**Keywords:** Cancer-associated fibroblasts, Extracellular vesicles, Cancer microenvironment, Intercellular communication, Non-coding RNA, Exosomes

## Abstract

Intercellular communication plays an important role in cancer initiation and progression through direct contact and indirect interactions, such as via secretory molecules. Cancer-associated fibroblasts (CAFs) are one of the principal components of such communication with cancer cells, modulating cancer metastasis and tumour mechanics and influencing angiogenesis, the immune system, and therapeutic resistance. Over the past few years, there has been a significant increase in research on extracellular vesicles (EVs) as regulatory agents in intercellular communication. EVs enable the transfer of functional molecules, including proteins, mRNAs and microRNAs (miRNAs), to recipient cells. Cancer cells utilize EVs to dictate the specific characteristics of CAFs within the tumour microenvironment, thereby promoting cancer progression. In response to such “education” by cancer cells, CAFs contribute to cancer progression via EVs. In this review, we summarize experimental data indicating the pivotal roles of EVs in intercellular communication between cancer cells and CAFs.

## Background

Although cancer cells arise from genetic mutations in normal cells, the malignant phenotypes of tumours are influenced by their surrounding tumour microenvironment (TME) [1]. The TME comprises various cellular components, including fibroblasts, endothelial cells, and inflammatory cells, as well as noncellular components, such as the extracellular matrix [1]. Accumulating evidence has demonstrated that among these TME components, cancer-associated fibroblasts (CAFs) modulate tumour metastasis and tumour mechanics and influence angiogenesis, the immune system, and therapeutic resistance [2]. Although CAFs are complex and include subtypes with protumorigenic and antitumour effects, several reports have indicated that CAFs are potential therapeutic targets for cancer treatment [2, 3]. Direct cell-to-cell contact and humoral factors such as cytokines and chemokines are considered the principal lines of communication between cancer cells and CAFs. However, recent studies have revealed that extracellular vesicles (EVs) are also regulatory players in such communication.

EVs are vesicles enclosed by a lipid bilayer membrane that include various bioactive molecules, such as proteins, mRNAs, metabolites, and microRNAs (miRNAs). Historically, EVs were initially considered “garbage bins” for exporting unnecessary intracellular components [4]. However, in 2007, Valadi et al. proposed that mRNAs packaged in EVs can be utilized for intercellular communication upon translation in recipient cells [5]. This discovery accelerated research on the role of EVs as novel intercellular communication tools. In particular, over the past ten years, there has been a striking expansion of EV research in cancer-related fields [6]. Various cancer-related factors packaged into EVs are transferred to recipient cells within proximal sites and distal metastatic niches. Intriguingly, Hoshino et al. indicated that integrin marks on EV surfaces could be considered a “zip-code” for delivery to specific organs [7], suggesting that cancer cells utilize EVs to confer specific features on cells in distal organs to support metastasis. In addition, cells within the TME, particularly CAFs, also regulate tumour progression, such as metastasis, by transferring EVs to cancer cells [6]. These findings imply that EVs derived from cancer cells and host-derived CAFs are involved in the formation of a suitable TME that promotes cancer progression.

In addition to the striking functions of EVs in intercellular communication, these vesicles can be detected in various body fluids, such as blood, saliva, and urine [8]. Therefore, EVs are attractive targets for improving cancer treatment and diagnosis. How do cancer cells utilize EVs to dictate the function and phenotypes of surrounding TME components to support cancer progression? This review will focus on recent advances in the interplay between cancer cells and CAFs and summarize the current knowledge regarding the functional role of EVs in the interplay between cancer cells and fibroblasts within the TME.

## Nomenclature of extracellular vesicles

There are various subtypes of EVs with differential components and biogenesis processes. They are traditionally classified into exosomes, ectosomes, and apoptotic bodies based on origin and size [4, 9]. Exosomes originate from the exocytosis of multivesicular bodies (MVBs) [9, 10]. Ceramide-dependent pathways and endosomal sorting complex required for transport (ESCRT) machinery partially regulate exosome release. Exosomes can contain the following proteins: membrane transport and fusion proteins (GTPase, annexins, and flotillin), tetraspanins (CD9, CD63, and CD81), heat shock proteins (Hsc70 and Hsp90), MVB-related proteins (Alix and TSG101), lipid proteins and phospholipases. Microvesicles are generated directly from budding of the plasma membrane [11]. Microvesicles can be enriched in some lipid components and phosphatidylserine [12]. Apoptotic bodies are generated during apoptosis. Thus, they contain intracellular fragments such as organelles, membranes, and cytosolic and nuclear fragments [13, 14]. However, it is still difficult to distinguish various types of EVs because of the lack of specific protein markers for each subtype. Thus, the expert consensus in EV research encourages the use of operational names unless specific and reliable EV markers of subcellular origin can be established [9]. (a) Physical characteristics, such as the size or density of EVs: small EVs (sEVs: < 100 nm or < 200 nm) and large and/or medium EVs (m/lEVs: > 200 nm). (b) Biochemical composition, such as positivity of staining for CD63, CD81, and Annexin A5. (c) Description of conditions or cells of origin, such as from podocytes and apoptotic bodies. In this review, to avoid confusion, we use the term EVs for all subtypes of membrane vesicles in the extracellular space rather than specific terms such as exosomes.

## What are CAFs?

To discuss the functional role of EVs in cancer-CAF interactions, we first need to define CAFs. CAFs are fibroblasts within the tumour stroma that can originate from many cell types, including resident fibroblasts, adipocytes, bone marrow-derived mesenchymal stem cells (BM-MSCs), and endothelial cells. The subtypes of CAFs include myofibroblast-like phenotypes similar to the features of activated fibroblasts during wound healing [15]. Many researchers, therefore, utilize adopted activated fibroblast markers such as alpha-smooth muscle actin (α-SMA) for the detection of CAFs [15]. However, recent promising studies and single-cell technologies have indicated that CAFs comprise diverse subtypes with distinct characteristics [16, 17]. Thus, conventional markers of CAFs are likely insufficient to detect all CAF subtypes within tumour tissue [18]. These findings are consistent with the fact that normal fibroblasts are also heterogeneous and lack unique markers that are not expressed in other cell types [2]. Although the precise definition of CAFs is still challenging, CAFs are practically considered morphologically elongated cellular components that are negative for epithelial, endothelial and leukocyte markers within tumour tissues [2]. To exclude the possibility of cancer cells undergoing epithelial–mesenchymal transition (EMT), CAFs are also defined as cells lacking genetic mutations found within the cancer cells [2].

The origin of CAFs and the signalling that mediates CAF heterogeneity remain controversial. Given the diverse subtypes in the normal fibroblast population, CAF subtypes may reflect their original tissue background. Indeed, in breast cancer, Raz et al. demonstrated that PDGFR-α-positive and PDGFR-α-negative subsets of CAFs are generated from BM-MSCs and resident fibroblasts, respectively [19]. They utilized a unique in vivo model involving BM implantation from GFP-expressing mice to MMTV-PyMT transgenic mice to track the cellular fate of BM-MSCs within the tumour mass. This study showed that BM-MSC-derived CAFs are negative for PDGFR-α expression and enhance angiogenesis and tumour growth. In contrast, in pancreatic ductal adenocarcinoma (PDAC), Tuveson's group showed that pancreatic stellate cells differentiate into two subsets of CAFs, inflammatory CAFs (iCAFs) and myofibroblastic CAFs (myCAFs) [20]. They also showed that IL-1 signalling induces iCAFs, but TGF-β signalling antagonizes this pathway, leading to myCAF differentiation [21]. Notably, these CAF subtypes are likely responsible for distinct functions for cancer progression, such as immunomodulatory functions and extracellular matrix (ECM) remodelling [19, 20]. These findings collectively imply that tissue environmental background and cancer-derived factors determine CAF complexity.

Diverse functions of CAFs in tumour progression have been reported. One substantial function of CAFs is as a source of various types of growth factors and chemokines, such as transforming growth factor-beta (TGF-β) [15, 22], CXC-chemokine ligand 12 (CXCL12)/SDF-1 [23], and HGF [24]. These CAF-derived factors promote an aggressive phenotype of cancer cells with invasive and metastatic behaviour, including the induction of EMT. The CD10 and GPR77 double-positive CAF subset secretes IL-6 and IL-8, sustaining cancer stemness and promoting chemoresistance [25]. In addition to direct effects on cancer cell behaviours, CAFs also generate VEGF to induce the angiogenesis of endothelial cells. CAFs also produce IL-6 and CXCL9 and regulate the immunosuppressive or immuno-promoting functions of inflammatory cells. The newly identified CAF subtype, antigen-presenting CAFs (apCAFs), can mediate CD4 + regulatory T-cell activation and CD8 + T-cell suppression [26]. These findings indicate that CAFs can mediate the function of both cancer cells and surrounding stromal cells within the TME.

Another essential function of CAFs is remodelling the extracellular matrix within the TME. CAFs produce ECM-related molecules, such as collagens and metalloproteases, which lead to the deposition and remodelling of the ECM. This remodelled ECM causes increased tissue stiffness, triggering proliferation signals in cancer cells. The increased tissue stiffness also promotes blood vessel collapse, reducing drug delivery. Matrix stiffening within tumour tissue also enhances the activation of YAP in normal fibroblasts and CAFs, which is involved in CAF generation and maintenance [27]. In addition, the metalloprotease produced by CAFs generates migratory tracks that allow cancer cell invasion [28]. Since CAFs can mediate the migration of cancer cells via E-cadherin/N-cadherin interactions [29], CAFs may be able to guide cancer cell invasion.

## Cancer-derived EVs can dictate preferable CAF characteristics for cancer progression

Over the past few years, there has been an increasing number of reports regarding the functional interplay of EVs in communication between cancer cells and CAFs (Tables 1 and 2). In this review, to better understand the intercellular crosstalk between cancer cells and CAFs via EVs, we focused on the papers that investigated EV components and revealed their functions on the recipient cells.

**Table 1:** Cancer-derived EV functions on CAF generation and property

Contents of cancer-derived EVs	Tumour type	EV donor (From)	EV recipient (To)	Functions of EVs on the CAF properties	Reference
**Proteins**		From	To		
Integrins	Breast cancer Pancreatic cancer	Cancer cells	CAFs	Up-regulates S100 gene expressions and promote cell qrowth and migration	[7]
TGF-β	Bladder cancer			Generate CAFs by activating SMAD-dependent pathway	[72]
Integlin αv and β1	Breast cancer			Integrin αv and β1 are enriched in CD63-positive EVs and induce CAF-like phenotypes in the fibroblasts. Galectin-3 might regulate the loading integrin αv and β1 in EVs.	[176]
ITGB4	Breast cancer			Induces BCL2 interacting protein 3 like (BNIP3L)-dependent mitophagy and glycolysis.	[33]
Sphingosine 1	Breast cancer			Sphingosine 1 drived from cancer cells may stimulate ERK-1/2 signalling and DNA synthesis	[73]
Survivin	Breast cancer			Generate CAFs with myofibroblastic features through inducing SOD1 expression to promote tumour proliferation and metastasis	[74]
Wnt2B	Cervical cancer			Generate CAFs through activating Wnt/β-catenin signalling	[75]
HSPC111	Colorectal cancer			Reprogramming lipid metabolism in CAFs to promote cancer metastasis.	[180]
TIMP-1	Colorectal cancer			Cancer-derived EVs transfer TIMP-1 to induced ECM remodelling in the fibroblasts.	[178]
LMP1	Epstein-Barr virus (EBV)-associated Nasopharyngeal carcinoma			Generate CAFs through NF-κB signalling and change aerobic glycolysis and autophagy in CAFs	[76]
PKM2	Gastric cancer			Generate CAFs by PKM2 nuclear translocation inducing NF-κB signalling	[77]
TGF-β	Head and neck squamous cell carcinoma			Generate CAFs through activation canonical TGF-β signalling pathway	[32]
PKM2	Hypoxic resistant lung cancer cells			Induce metabolic reprogramming in CAFs	[78]
IGF2	Liver cancer			Fluid shear stress-induced cancer cell medium promoted the activation and proliferation of CAFs via activating PI3K/AKT signaling pathway.	[172]
α-SMA	Lung cancer			Cancer-derived EV transfer α-SMA in both lung cancer cell line and fibroblasts. These EVs also promote cell proliferation and inhibit apoptosis.	[79]
TGF-β	Malignant ascite from gastriccancer and ovarian cancer			TGF-β in EVs may induce CAF phenotypes in peritonieal mesothelial cells	[80]
HSP90 and p-IKKα/β complex	Melanoma			Promote the proangiogenic capacity via activating NF-κB signalling to induce CXCL1 expression in CAFs.	[173]
TGF-β	Mesothelioma Colorectal cancer Prostate cancer Bladder cancer			Triger the myofibroblast differen-tiation	[30]
FAP and EBV-encoded latent membrane protein 1 (LMP1)	Nasopharyngeal carcinoma			Generate CAFs via enhancing YAP1 signalling and increasing FAP expression.	[177]
COL6A1	Osteosarcoma			COL6A1-positive EVs generate CAFs expressing interleukins, α-SMA, and TGF-β	[81]
Gain-of-function p53	Ovarian cancer			Instigate CAF phenotypes in fibroblasts through the Nrf2-dependent pathway	[39]
Annexin A1	Pancreatic cancer			Induce myofibroblasts features in the fibroblasts and endothelial cells	[82]
Lin28B	Pancreatic cancer			Generate CAFs from pancreatic stellate cells through activating let-7/HMGA2/PDGFRβ axis.	[83]
Hyal1	Prostate cancer			Stimulate fibroblast chemotaxis by the increased adhesion and activating FAK signalling.	[84]
TGF-β	Prostate cancer			Triger the myofibroblast differen-tiation and promote cancer growth and angiogenesis	[31]
C-terminal Dsg2	Squamous cell carcinoma			Dsg2-positive EVs activate Erk1/2 signalling and promote cell proliferation in fibroblasts.	[85]
**Coding RNAs**		From	To		
Inflammation-inducing mRNAs	Melanoma	Cancer cells	CAFs	Induce CAF subtype with inflammatory signatures in within metastatic niche	[47]
**non-coding of transposable RNAs**		From	To		
miR-105	Breast cancer	Cancer cells	CAFs	Reprogram glucose and glutamine metabolism to fuel adjacent cancer cells	[34]
miR-105 and miR-204	Breast cancer			Cancer-derived EVs might transfer miR-105 and miR-204 and suppress RAGC expression in fibroblasts.	[175]
miR-122	Breast cancer			Down-regulates glucose consumption of fibroblasts	[63]
miR-125b	Breast cancer			Generates CAFs from resident fibroblasts through targeting TP53INP1 expression	[43]
miR-130b-3p	Breast cancer			Generate CAFs via targeting SPIN90 in fibroblasts and facilitate cancer progression.	[179]
miR-185-5p, miR-652-5p, and miR-1246	Breast cancer			Three miRNAs in cancer-derived EVs can be involved in the induction of CAF phenotypes in normal fibroblasts.	[171]
miR-370-3p	Breast cancer			Induce fibroblast activation through CYLD/NF-κB signalling and promote cancer progression	[86]
Decreased miR-34c	Cholangiocarcinoma			Enhancing Wnt1 expression in fibroblasts and generate CAFs. miR-34 directly target Wnt1 expression.	[87]
miR-146a	Chronic lyphocytic leukaemia			Generate CAFs by targeting USP16 expression in fibroblasts	[88]
miR-10a	Colorectal cancer (SW480 cell line)			Inhibit migration and expression of IL-6 and IL-8 expression in fibroblasts	[89]
miR-1249-5p, miR-6737-5p, and miR-6819-5p	Colorectal cancer			EVs derived from cancer cells with p53 shRNA genrate CAFs. miRNAs in EVs may contribute to induction of CAF phenotype in fibroblasts.	[44]
miR-146a-5p and miR-155-5p	Colorectal cancer			Generate CAFs via targeting SOCS1 and ZBTB2 to activate JAK2-STAT3/NF-κB signalling. CXCL12/CXCR7 is associated with these miRNA expressions in cancer cells.	[181]
miR-200 family	Colorectal cancer			CRC cells with an epithelial phenotype but not a mesenchymal phenotype secrete miR-200 family members via EVs to attenuate TGF-β-mediated CAF features by targeting ZEB1 in normal fibroblasts.	[45]
miR-4534	Colorectal cancer			Suppress autophagy to induce CAF phenotypes in fibroblasts. EV miR-4534 targets ATG2B expression.	[182]
miR-27a	Gastric cancer			Generate CAFs to support cancer migration and invasion.	[90]
Several miRNAs including miR-193b	Gastric cancer			Induce CAF subtype with inflammatory signatures	[46]
miR-192, miR-215	Head and neck squamous cell carcinoma			Generate CAFs through targeting CAV1 to induce TGF-β/SMAD signalling	[91]
miR-9-5p	Head and neck squamous cell carcinoma			HPV-positive head and neck squamous cell carcinoma cells secrete miR-9-5p via EVs. EV miR-9-5p suppresses NOX4 expression to inhibit the induction of TGF-β-mediated CAF phenotype in fibroblasts.	[174]
miR-21	Hepatocellular carcinoma			Generate CAFs from hepatocyte stellate cells through activating PDK1/Akt signalling	[92]
miR-1247-3p	Hepatocellular carcinoma			Generate CAFs expressing inflammatory genes through targeting B4GALT3 to activate β1 integlin/NF-κB signalling	[49]
miR-181d-5p	Hepatoma cell			May induce CAF state via targeting SOCS3 expression in bone-marrow stem cells (BMSCs)	[93]
miR-3473b	Lewis lung carcinoma			Generate inflammatory gene expressing CAFs through activationg NF-κB singnalling	[94]
miR-142-3p	Lung cancer			EVs derived from cancer cells with miR-142-3p over-expression generate CAFs via non-canonical TGF-β signalling	[96]
miR-210	Lung cancer			Generate CAFs expressing proangiogenic factors through activating JAK2/STAT3 signalling	[95]
lncRNA Gm26809	Melanoma			Generate CAF properties in NIH3T3 fibroblasts	[97]
miR-155	Melanoma			Generate CAFs expressing proangiogenic factors through inhibiting SOCS1 to activate JAK2/STAT3 signalling	[99]
miR-155 and miR-210	Melanoma			Increase aerobic glycolysis and decrease oxidative phosphorylation in fibroblasts.	[98]
miR-375	Merkel cell carcinoma			Generate CAFs through targeting RBPJ and p53 expression	[100]
miR-21	Mouse melanoma cell			Promote the invasion activity of fibroblasts through targeting TIMP-3 expression	[101]
lncRNA (LncRNA-CAF)	Oral squamous cell carcinoma			Stimulate IL-33 expression and CAF phenotypes in fibroblasts to influence CAF generation from other surrounding fibroblasts and promotes tumour growth	[102]
miR-630	Ovarian cancer			Generate CAFs through targeting KLF6 and activating NF-κB signalling pathway	[103]
miR-155	Pancreatic cancer			Induce cancer-associated fibroblast like phenotype through repressing TP53P1	[104]
Several miRNAs including miR-1246 and miR-1268	Rhabdomyosarcoma			Rhabdomyosarcoma-derived EVs promote cell growth and stimulate angiogenic capacities in fibroblasts	[105]
**Others**		From	To		
Not investigated	Bladder cancer	Cancer cells	CAFs	Cancer-derived EVs induce inflammatory CAFs (iCAFs)	[48]
miR-1246, TGF-β, β-catenin, IL-6, p-STAT3	Colorectal cancer			Cancer-derived EVs generate CAFs. But the precise mechanism of how molecules in EVs induce CAF signatures in fibroblasts is not addressed.	[108]
Not investigated	Colorectal cancer			Stimulate migration capacity of CAFs via activating Rho-Fak signalling	[106]
Not investigated	Colorectal cancer			Cancer-derived EVs generate CAFs to acquire the capacity to invade matrix and to support cancer invasion.	[107]
Not investigated	Experimentally induced cancer stem cells (Piwil2-CSC)			Generate CAFs and enhance cell migration and invasion in CAFs.	[109]
Not investigated	Gastric cancer			Generate CAFs from pericytes through activating PI3K/AKT and MEK/ERK signalling pathways	[110]
Not investigated	Gastric cancer			Generate CAFs through canonical TGF-β signalling pathway	[111]
Not investigated	Lung cancer			Mediate immunomodulate effect via inducing PD-L1	[112]
Not investigated	Lung cancer			Cancer cells with TP53 mutation mediate the integrin trafficking in the fibroblasts via EVs and promote the deposition of invasive ECMs	[38]
Not investigated	Ovarian cancer			Cancer-derived EVs instigate cell adhesion and migration capacity in CAFs.	[67]
Not investigated	Prostate cancer			Cancer-derived EVs stimulate prometastatic factors including brain-derived neurotrophic factor and CXCL12.	[113]
Not investigated	Salivary adenoid cystic carcinoma			Induce the capacity to enhance cancer invasion and NGF expression in human periodontal ligament fibroblasts.	[114]

**Table 2:** CAF-derived EV functions on cancer cell property

Contents of CAF-derived EVs	Tumour type	EV donor (From)	EV recipient (To)	Functions of CAF-derived EVs on cancer progression	References
**Proteins**		From	To		
CD81	Breast cancer	CAFs	Cancer cells	Enhance cancer motility and metastasis through activating Wnt-planar cell polarity (PCP) signalling pathway	[50]
Extracellular matrix proteins and ADAM10	Breast cancer			Induce aldehyde dehydrogenase expressionin cancer cells through Notch activation and enhance motility through the GTPase RhoA.	[115]
Wnt10b	Breast cancer			CAFs with low p85α expression transfer Wnt10b via EVs and promote cancer progression.	[116]
Amphiregulin	Colorectal cancer			TGF-β-induced CAF model secrete Amphiregulin via EVs and promote cell proliferation in EGF-dependent patient derived organoids	[118]
Wnt3a	Colorectal cancer			Expansion of cancer stem cell to enhance chemoresistance	[117]
Sonic Hedgehog (Shh)	Esophageal squamous cell carcinoma			Promote cancer cell proliferation. CAF-derived EVs may transfer Shh to enhance tumour growth.	[119]
Annexin A6	Gastric cancer			Activate FAK-YAP signalling through stavilizing integrin β1 to enhance drug resistance.	[120]
CD9	Gastric cancer			CAFs secrete CD-9 positive EVs and stimulate diffuse-type GC migration.	[51]
INHBA and THBS1/2	Gastric cancer			INHBA and THBS1/2 are associated with aggressive property of gastric cancer. HSF-1, master regulator of these molecules, may contribute to the loading of these molecules in MEF- and CAF-derived EVs.	[121]
Gremlin-1	Hepatocellular carcinoma			Promote EMT and sorafenib resistance via activating Wnt/β-catenin signalling pathway.	[200]
SLPI	Ovarian cancer			Promote cancer progression by activating PI3K and MAPK signalling pathways.	[194]
TGF-β1	Ovarian cancer			Promote EMT in cancer cells through inducing canonical TGF-β singalling.	[122]
Annexin A6	Pancreatic cancer			ANXA6- and CD9-double positive CAF-derived EVs facilitate p38 MAPK signal activation to enhance migratory ability in PDAC cells.	[52]
Galectin-1	Prostate cancer Pancreatic cancer Melanoma			CAF-derivved galectin-1 may contribute to cancer migration.	[123]
**Coding RNAs**		From	To		
SNAI1 mRNA	Lung cancer	CAFs	Cancer cells	Transfer SNAI1 mRNA and promote EMT in cancer cells.	[124]
**non-coding of transposable RNAs**		From	To		
LINC00355	Bladder cancer	CAFs	Cancer cells	Function as a sponge of miR-15a-5p and increase HMGA2 expression to promote cancer progression	[125]
LINC00355	Bladder cancer			Promote cancer cell proliferation.	[126]
Decreased miR-1-3p	Breast cancer			miR-1-3p expression is decreased in CAF-derived EVs. These CAF-derived EVs promote cancer progression. miR-1-3p targets GLIS1 expression in cancer cells.	[133]
Decreased miR-4516	Breast cancer			miR-4516 expression is decreased in CAF-derived EVs and also promote cancer progression. miR-4516targets FOSL1 to promote cancer progression.	[131]
Decreased miR-7641	Breast cancer			miR-7641 expression is decreased in CAF-derived EVs. These CAF-derived EVs can induce cancer stemness and metabolic reprogramming.	[132]
lncRNA SNHG3	Breast cancer			CAF-derived SNHG3 act as sponge for miR-330-5p expression to increase PKM protein expression results in metabolic reprogramming in cancer cells.	[62]
miR-16 and miR-148a	Breast cancer			FAK-null CAFs suppress cancer metastasis, and this CAF-derived EVs can partially contribute to the reduced tumour cell activities and metastasis.	[129]
miR-181d-5p	Breast cancer			Promote cancer cell migratrion and invasion through targeting CDX2 and HOXA5 to induce EMT in cancer cells.	[130]
miR-18b	Breast cancer			Promote cancer invasion and metastasis by targeting Transcription Elongation Factor A Like 7 (TCEAL7) expression in cancer cells.	[195]
miR-22	Breast cancer			CD63-positive CAF-derived EVs espress miR-22 and promote Tamoxifen resistance in cancer cells through targeting SFRS1 expression.	[58]
miR-3613-3p	Breast cancer			Promote cancer cell proliferation through targeting SOCS2 expression in cancer cells	[128]
miR-500a-5p	Breast cancer			Promote cancer proliferation and metastasis through targeting USP expression	[127]
miR-92	Breast cancer			Target directly the expression of LAT2 and induces PD-L1 expression through YAP activation in breast cancer cells.	[58]
Non-coding of transposable RNAs	Breast cancer			Stimulate RIG-I recognition to activate STAT1 in cancer cells.	[53]
miR-1323	Cervical cancer			Promote radioresistance via targeting PABPN1 and activating Wnt/β-catenin signalling pathway.	[198]
circN4BP2L2	Colorectal cancer			Promote cancer cell proliferation and metastasis. circN4BP2L2 in EVs targets miR-664b-3p to induce HMGB3 expression.	[188]
lncRNA CCAL	Colorectal cancer			Promotes Oxaliplatin resistance in CRC cells via stabilizing human antigen R (HuR) mRNA to increase β-catenin expression.	[56]
lncRNA H19	Colorectal cancer			Promote Cancer stemness and chemoresistance through targeting miR-141 expression and activating β-catenin signalling	[137]
lncRNA LINC00659	Colorectal cancer			Act as an RNA sponge for miR-342-3p expression to induce ANXA2 expression and promote EMT in cancer cells.	[142]
LncRNA SNHG3	Colorectal cancer			Promote cell proliferation via targeting miR-34b-5p to induce HOXC6 expression in cancer cell.	[196]
miR-135b-5p	Colorectal cancer			Promote tumour angiogenesis via targeting FOXO1 expression in cancer cell.	[189]
miR-135b-5p	Colorectal cancer			Might promote tumour angiogenesis and cancer cell proliferation via TXNIP expression.	[202]
miR-17-5p	Colorectal cancer			Promote cancer progression through targeting RUNX3 to activate MYC/TGF-β axis in cancer cells	[134]
miR-181-5p	Colorectal cancer			Promote 5-FU resistance via targeting neurocalcin δ (NCALD) expression in colorectal cancer cells.	[193]
miR-21	Colorectal cancer			Promote liver metastasis via transfering miR-21 and influence cell proliferation and chemoresistance.	[138]
miR-224-5p	Colorectal cancer			Involve in cancer progression through targeting SLC4A4 expression.	[141]
miR-24	Colorectal cancer			Enhance methotrexate resistance of cancer cells via targeting CDX2 to regulate HEPH expression.	[139]
miR-590-3p	Colorectal cancer			Promote the radioresistance in cancer cells through targeting CLCA4 to activate PI3K/AKT signalling pathway and reduce expressions of cleaved-caspase 3 and cleaved-PRAP.	[140]
miR-92a-3p	Colorectal cancer			Promote cancer metastasis and chemotherapy resistance through targeting FBXW7 and MOAP1 expression	[136]
miR-93-5p	Colorectal cancer			Targeting FOXA1 and induce TGFB3b and promote radioresistance	[135]
Decreased miR-320a	Endometorial cancer			miR-320a expression is decreased in CAF-derived EVs and promote cancer progression. miR-320a target HIF1α expression in cancer cells.	[143]
Decreased miR-148b	Endometorial cancer			miR-148b expression is decreased in CAF-derived EVs to enhance cancer metastasis through inducing EMT. miR-148b in EVs may targeting DNMT1 expression.	[144]
lncRNA NEAT1	Endometorial cancer			Act as an RNA sponge for miR-26a/b-5p expression to induce YKL-40 expression via STAT3 signalling and enhance cancer progression.	[145]
LINC01410	Esophageal cancer			Promote EMT via targeting miR-122 and inducing PKM2 expression.	[199]
miR-21	Esophageal squamous cell carcinoma			CAF-derived miR-21 cooperates with IL-6 to induce monocytic myeloid-derived suppressor cells (M-MDSCs), resulting in cisplatin resistance regulation in cancer cells.	[146]
Decreased miR-34	Gastric cancer			miR-34 expression is decreased in CAF-derived EVs and promote cancer progression.	[147]
lncRNA circ_0088300	Gastric cancer			Act as an RNA sponge for miR-1305 expression to promote cancer progression. KHDRBS3 contributes to circ_0088300 transfer via CAF-derived Evs.	[150]
miR-199a-5p	Gastric cancer			Promote cancer progression by targeting FKBP5 expression and activating mTORC1 signalling in cancer cells.	[201]
miR-522	Gastric cancer			Suppress ferroptosis via targeting ALOX15 expressiion and promote chemoresistance. hnRNPA1 and USP7 are associated with miR-522 secretion via CAF-derived EVs.	[148]
MMP-11 and Decreased miR-139	Gastric Cancer			Transfer MMP-11 to promote cancer cell progression. miR-139 expression is decreased in CAF-derived EVs and also promote cancer progression. miR-139 may target MMP11.	[149]
Decreased miR-3188	Head and neck cancer			miR-3188 expression is decreased in CAF-derived EVs to promote cell proliferation and inhibit apoptosis in cancer cells. EV-derived miR-3188 targets BCL2 expression.	[151]
miR-196a	Head and neck cancer			Promote cisplatin resistance via targeting CDKN1B and ING5 expression.	[152]
Decreased miR-150-3p	Hepatocellular carcinoma			miR-150-3p expression is decreased in CAFs and their EVs to promote cancer cell progression.	[153]
Decreased miR-29b	Hepatocellular carcinoma			miR-29b expression is decreased in CAF-derived EVs and promote cancer migration and invasion. miR-29b may target DNMT3b to modulate EMT in cancer cells.	[154]
Decreased miR-320a	Hepatocellular carcinoma			miR-320a expression is decreased in CAF-derived EVs. These CAF-derived EVs promote cancer progression. miR-320a targets PBX1 expression in cancer cells.	[155]
Decreased let7a-5p	Lung cancer			Sulfonylurea receptor 1 (SUR1) expressing cancer cells decreased let-7a-5p expression in their EVs to induce CAFs. Mechanistically, let-7a-5p targets TGFBR1 to inactivate the TGF-β signalling in fibroblasts.	[184]
lncRNA OIP5-AS	Lung cancer			Export OIP-AS to suppress miR-142-5p and induce PD-L1 expression. EVs can be involved in immune tolerance of tumour.	[183]
LncRNA SNHG12	Lung cancer			Promote cisplatin resistance by binding to HuR to facilitate RNA stability and XIAP expression.	[187]
lncRNA TUG1	Lung cancer			Promote cancer cell migration, invasion, and glycolysis. TUG1 in EVs may target miR-524-5p to induce SIX1 expression.	[185]
miR-103a-3p	Lung cancer			Suppress apoptosis and promote cisplatin resistance through targeting Bak1 expression. Pum2 contributes to miR-103a loading into CAF-derived EVs.	[159]
miR-130a	Lung cancer			Cisplatin-induced miR-130a is transferred by CAF-derived EVs and promotes chemoresistance in cancer cells. PUM2 contributes to miR-130a packaging into CAF-derived EVs.	[158]
miR-20a	Lung cancer			Promote cancer cell proliferation and cisplatin resistance via targeting PTEN expression in non-small cell lung cancer cells.	[192]
miR-210	Lung cancer			Promote cancer cell migration and invasion through targeting UPF1 to induce EMT in cancer cells	[156]
miR-369	Lung cancer			Promote cancer cell migration and invasion through targeting NF1 and mediate MAPK signalling pathway	[157]
miR-4717-5p	Malignant lymphoma			Induce gemcitabine resistance through targeting ENT2 expression in cancer cells.	[55]
Decreased miR-34a	Oral squamous cell carcinoma			Promote cancer cell proliferation and metastasis through AKT/GSK-3β/β-catenin signalling. miR-34a can target AXL in cancer cells.	[161]
miR-382-5p	Oral squamous cell carcinoma			Promote cancer cell migration and invasion. miR-382-5p contributes to EV-mediated cancer progression.	[160]
miR-1228	Osteosarcoma			Promote cancer migration and invasion through targeting SCAI.	[162]
miR-21 isomiR	Ovarian cancer			Confer the chemo-resistance through targeting APAF1	[163]
miR-98-5p	Ovarian cancer			Promote cisplatin resistance via targeting CDKN1A.	[164]
miR-106b	Pancreatic cancer			Gemcitabine induce miR-106a in CAFs and their EVs. CAF-derived miR-106a can promote cancer cell proliferation.	[165]
miR-331-3p	Pancreatic cancer			Promote cancer progression via targeting SCARA5 expression in PDAC cells.	[191]
miR-92a-3p, miR-181a-5p, miR-222-3p, miR-221-3p, miR-21-5p	Pancreatic cancer			CAFs might transfer miRNAs in PDAC cells via EVs and promote gemcitabine resistance. miR-92a-3p could target PTEN expression in PDAC cells.	[190]
miR-1290	Prostate cancer			Promote EMT and metastasis via targeting GSK3β expression in cancer cell.	[197]
miR-146a-5p	Prostate cancer			Promote cancer metastasis. Treatment of Dihydrotestosterone (DHT) decrases miR-146a-5p in CAF-derived EVs and attenuate cancer promoting function.	[186]
miR-409	Prostate cancer			Promote epithelial-mesenchymal transition through repression of tumor suppressor genes such as Ras suppressor 1 and stromal antigen 2	[166]
miR-224-5p	Renall cell carcinoma			Contribute to cancer progression	[167]
**Metabolites**		From	To		
mitochondorial DNA (mtDNA)	Breast cancer	CAFs	Cancer cells	Promote estrogen receptor-independent oxidative phosphorylation restore in cancer-stem like cells and increase self-renewal capacity in these cells.	[168]
Metabolites including amino acids and lipids	Prostate cancer			Affect the metabolic properties in prostate cancer cells	[60]
**Others**		From	To		
RN7SL1	Breast cancer	CAFs	Cancer cells	Acting as damage-associated molecular patterns (DAMPs) and activating RIG-I in the recipient cancer cells	[54]
Protein binding, serine-type endopeptidase activity, signalling receptor and protein kinase binding-related molecules	Oral squamous cell carcinoma			CAF-derived EVs promote cancer invasion and apoptosis.	[169]
miR-146a and Snail mRNA	Pancreatic cancer			Gemcitabine induce the secretion of miR-146 and Snail mRNA via EVs and regulate survival and proliferation in cancer cells.	[170]

**Fig. 1:**
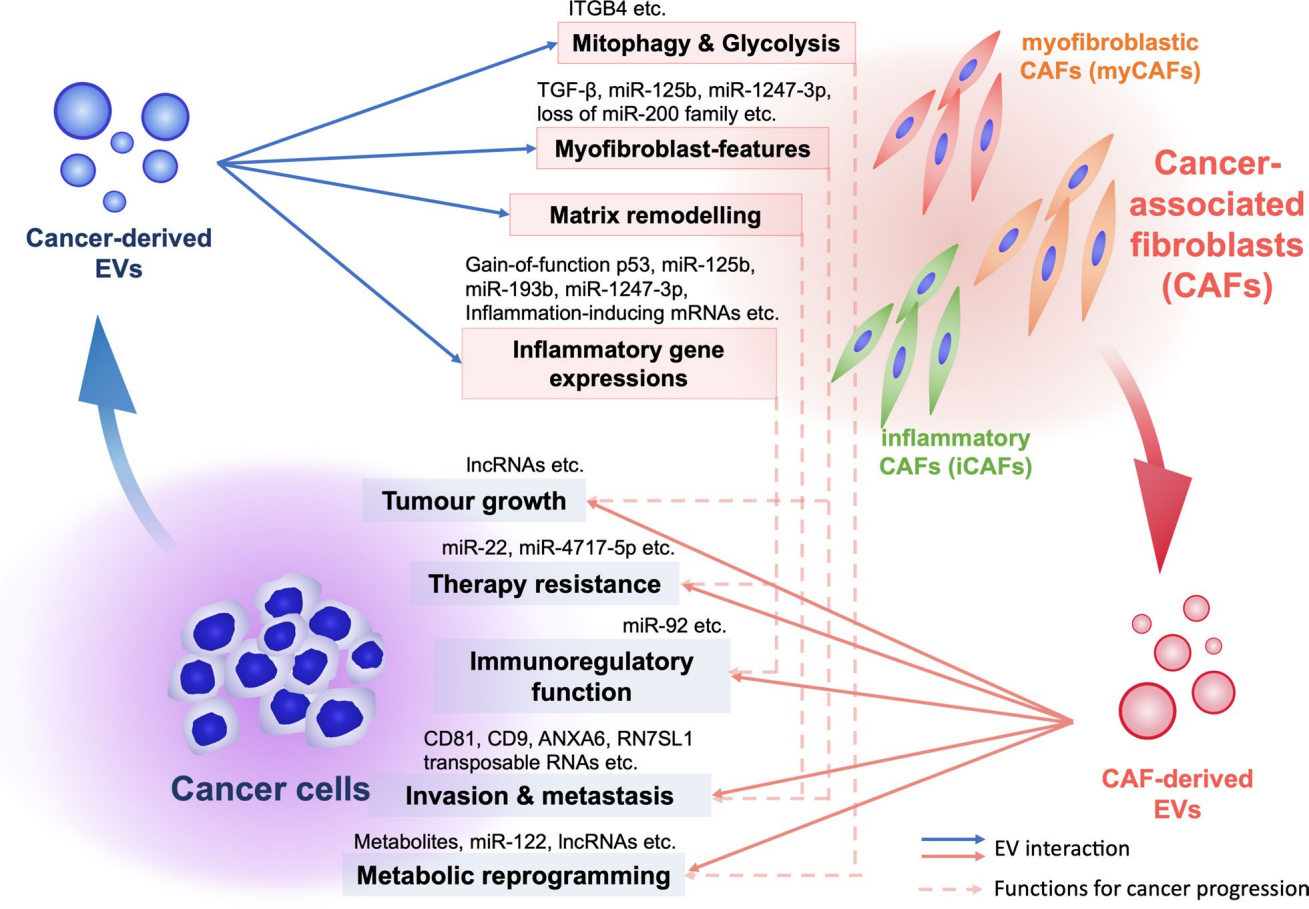
Summary of the intercellular crosstalk between cancer cells and CAFs via EVs. The interplay between cancer cells and cancer-associated fibroblasts (CAFs) generates a unique tumour microenvironment (TME) that supports cancer progression. Cancer cells utilize EVs to modify surrounding cells within the tumour microenvironment. Cancer cells secrete various bioactive molecules in EVs, which are transferred into the surrounding fibroblasts and CAFs; such molecules include transforming growth factor-beta (TGF-β), ITGB4, p53, mRNAs and miRNAs (Table 1). These cancer-derived EVs confer specific phenotypes, such as mitophagy and glycolysis, myofibroblast features, matrix remodelling, and inflammatory gene expression (blue arrows), to support cancer progressions (broken arrows, the functions for cancer progression) (Table 1). Cancer-derived EVs also induce distinct CAF subtypes and contribute to the heterogeneity of CAFs. On the other hand, to support cancer progression, CAFs utilize EVs to transfer various functional molecules, such as CD81, CD9, FAP, miRNAs, lncRNAs, transposable RNAs and metabolites. These molecules confer aggressive phenotypes on cancer cells (red arrows). However, some molecules are downregulated in CAFs and thus their transfer to cancer cells is prevented (Table 2). Interestingly, specific CAF subtype-derived EVs are also involved in cancer progression, suggesting that EV content may differ among CAF subtypes

Many researchers have reported how CAFs are generated by cancer cells. Both direct contact and indirect interaction between cancer cells and CAFs are essential to maintain CAF characteristics. Among these interactions, TGF-β signalling is a well-established factor for activating fibroblasts and generating CAFs [2]. In addition to these mechanisms, cancer cells utilize EVs to generate CAFs (Fig. 1 and Table 1). The first report on cancer-derived EVs related to CAF induction was published by Webber et al., who showed that TGF-β was expressed on the surface of EVs derived from prostate cancer and mesothelioma cell lines [30, 31]. TGF-β on these cancer-derived EVs triggered the TGF-β/SMAD3 signalling pathway in recipient fibroblasts and induced myofibroblast-like phenotypes, including the expression of α-SMA and fibroblast growth factor 2 (FGF2) [30]. Recently, Huang et al. also showed that TGF-β in EVs generates CAFs via a noncanonical fibronectin-dependent pathway [32]. These reports demonstrated that EVs are responsible for some of the signalling previously thought to be carried out by growth factors. In studying triple-negative breast cancer, Sung et al. utilized an experimental model expressing integrin beta 4 (ITGB4) to determine the effect of EVs on the properties of CAFs [33]. Cancer-derived EVs transfer ITGB4 and induce BCL2 interacting protein 3 like (BNIP3L)-dependent mitophagy and glycolysis in CAFs. They also showed that the overexpression of ITGB4 in CAFs promotes breast cancer cell proliferation, EMT, and invasion [33]. Yan et al. demonstrated the reprogramming of glucose and glutathione metabolism in CAFs induced by miR-105 transferred by metastatic breast cancer cell-derived EVs [34]. Targeting of MXI1 by miR-105 activates MYC, the essential driver of metabolic reprogramming in CAFs. Long non-coding RNAs (lncRNAs) are also transferred from cancer cells to fibroblasts. Since the exchange of metabolites and amino acids is one mechanism of interplay between cancer cells and CAFs [35–37], it is highly plausible that cancer cells utilize EVs to dictate metabolic reprogramming in CAFs and thus construct a preferable microenvironment for their progression.

Cancer cell properties such as TP53 gene mutation also affect EV components and have consequences on CAF generation [38–41]. Novo et al. demonstrated that TP53-mutant cancer cells mediate integrin trafficking in fibroblasts via EVs and promote the deposition of a pro-invasive ECM [38]. Although they did not focus on molecules encapsulated in EVs, this finding implies that EVs are involved in matrix remodelling within the TME via CAF generation and activation. Interestingly, Ma et al. showed the direct transfer of gain-of-function p53 proteins from cancer cells to fibroblasts via EVs. This gain-of-function p53 isoform induced a CAF phenotype in fibroblasts through the Nrf2-dependent pathway [39]. Since Nrf2 is the essential regulator of ECM production and deposition [42], transferring the gain-of-function p53 isoform to CAFs might involve a proinvasive ECM construction. In addition, Vu et al. found that miR-125b in breast cancer cell-derived EVs generates CAFs from resident fibroblasts [43]. Downregulation of TP53INP1 by EV-transferred miR-125b induced a CAF state in fibroblasts to promote tumour growth [43]. Given that aberrant p53 function can confer a CAF phenotype on fibroblasts [38, 39, 44], the expression status of the TP53 gene in both cancer cells and CAFs may reflect the features of CAF subtypes. Recently, Bhome et al. demonstrated that the EMT phenotype of colorectal cancer (CRC) cells influences CAF generation [45] CRC cells with an epithelial phenotype secrete miR-200 family members via EVs to attenuate TGF-β-mediated CAF features by targeting ZEB1 in normal fibroblasts. However, EVs derived from CRC cells with a mesenchymal phenotype contain less miR-200 family, thus allowing TGF-β-mediated CAF feature induction. An aggressive subtype of CRC based on the consensus molecular subtypes (CMSs) is associated with mesenchymal gene signatures. Their study provides one of the essential clues as to why cancer cells with aggressive phenotypes possess CAF-abundant stroma.

EVs also generate specific CAF subtypes from resident fibroblasts, promoting CAF heterogeneity. Our group reported that highly metastatic gastric cancer (GC) cells secrete EVs and induce the expression of chemokines such as IL-1 and IL-8 in stomach fibroblasts, which have similar properties as iCAFs [46]. Regarding the mechanism, these GC-derived EVs transport miRNAs, including miR-193b, to fibroblasts and induce chemokine expression. On the other hand, these GC cell-derived EVs do not have the capacity to regulate the expression of α-SMA and collagen, which are myCAF features. Notably, compared with GC cells with low metastatic potential, those with high metastatic potential can more strongly induce both α-SMA and chemokine expression in fibroblasts. These data suggest that cancer-derived EVs selectively confer iCAF-like features on fibroblasts. IL-8-expressing CAFs within the tumour stroma are closely associated with poor outcomes in patients with GC. These findings collectively imply that GC generates a protumorigenic microenvironment to support disease progression. Other research groups have also shown that cancer-derived EVs affect CAF heterogeneity [47, 48]. Goulet et al. showed that bladder cancer-derived EVs induce iCAF features in primary bladder fibroblasts [48]. Compared with TGF-β treatment, these cancer-derived EVs have a weaker effect on the induction of α-SMA in fibroblasts [48]. Although these researchers did not clarify the contribution of cancer-derived EVs to iCAF differentiation, they showed that EV-induced iCAFs express IL-6 and activate the STAT3 signalling pathway that leads to EMT in bladder cancer [48]. Lahav et al. demonstrated that metastatic melanoma cell-derived EVs activate proinflammatory signalling in fibroblasts within metastatic niches [47]. They also found that melanoma cell-derived EVs contain RNAs capable of instigating inflammatory signalling in fibroblasts, such as high-mobility group box 1 (Hmgb1), thymic stromal lymphopoietin (Tslp), and interferon regulatory factor 1 (Irf1) [47]. Interestingly, these EVs cannot induce myCAF features, suggesting that metastatic cancer cell-derived EVs may preferentially encourage iCAF features rather than myCAF features [49]. These findings indicate that EVs deliver messages from cancer cells, instigate various features in CAFs, and affect the feedback from CAFs for cancer progression.

## CAF-derived EVs change the properties of cancer cells during disease progression

Stromal fibroblasts “educated” by cancer cells also secrete EVs and establish intercellular communication that ultimately benefits cancer progression (Fig. 1 and Table 2). Luga et al. first reported the functional role of CAF-derived EVs in cancer progression. They showed that CD81-positive CAF-derived EVs activate the Wnt-planar cell polarity (PCP) signalling pathway and enhance breast cancer cell motility and metastasis [50]. CD81 on the surface of EVs may support the endocytic trafficking of Wnt11 in cancer cells. This mechanism mediated by CAF-derived EVs activates the Wnt-PCP pathway in cancer cells to drive invasive behaviour [50]. CD81, a tetraspanin family member protein, is a conventional marker of EVs [9]. Other tetraspanin family proteins may also support the internalization of CAF-derived EVs. Miki et al. showed that CAFs secrete CD-9-positive EVs and stimulate diffuse-type GC migration. In addition, they showed that a CD9 neutralizing antibody inhibits EV uptake by GC cell lines [51]. Consistent with this finding, Nigri et al. also demonstrated that CD9 neutralizing antibody impaired CAF-derived ANXA6-positive uptake by PDAC cells [52]. ANXA6 can interact with CD9 in CAFs, suggesting that CD9 might regulate EV-specific ANXA6 shuttling. ANXA6- and CD9-double-positive CAF-derived EVs facilitate p38 MAPK signal activation to enhance migratory ability in PDAC cells. Although these reports did not clarify how the suppression of CD9 expression inhibits EV uptake in cancer cells, they identified CD9 function as a mediator of the internalization of CAF-derived EVs into cancer cells.

CAF-derived EVs support the development of cancer cell therapeutic resistance. Minn's group reported the precise mechanisms by which breast cancer cells achieve therapeutic resistance through CAF-derived EVs [53, 54]. CAF-derived EVs contain transposable RNAs that stimulate RIG-I recognition to activate STAT1 in cancer cells. Activated STAT1 cooperates with juxtacrine-activated NOTCH3 to mediate NOTCH target gene transcription that supports resistance to chemotherapy and radiation [53]. They also found that NOTCH-MYC signalling induces RN7SL1 expression in CAFs. RN7SL1 generally possesses 5′ppp capping that is regulated by SRP9 and SRP14, which may prevent its recognition by RIG-I. However, NOTCH-MYC signalling in CAFs induces RN7SL1 without 5′ppp capping, which is encapsulated into CAF-derived EVs and acts as a damage-associated molecular pattern (DAMP) to activate RIG-I in recipient cancer cells [54]. Kunou et al. demonstrated the function of CAF-derived EVs in the gemcitabine resistance in malignant lymphoma [55]. These CAF-derived EVs suppress the expression of equilibrative nucleoside transporter 2 (ENT2) in lymphoma cells. They also found that miR-4717-5p is one of the most abundant miRNAs in CAF-derived EVs and directly targets ENT2 expression [55]. In colorectal cancer (CRC), Deng et al. showed that FAP-positive CAFs secrete lncRNAs via EVs [56]. These CAF-derived lncRNAs promote oxaliplatin resistance in CRC cells in vitro and in vivo by stabilizing human antigen R (HuR) mRNA to increase β-catenin expression. The FAP-positive CAF subset is associated with regulatory T-cell-mediated immunosuppression, which results in poor outcomes in breast cancer patients [57]. Thus, it is possible that the FAP-positive CAF subset also secretes functional EVs to facilitate the malignant behaviour of cancer cells. Indeed, Dou et al. reported the immunomodulatory functions of CAF-derived EVs [58]. Although they did not refer to CAF subsets, they showed that CAF-derived miR-92 directly targets LATS2 expression and induces PD-L1 expression through YAP activation in breast cancer cells [58].

The functional role of CAF subset-derived EVs in cancer progression was recently reported. Gao et al. identified CD63-positive CAF subsets using single-cell analysis of oestrogen receptor α (ERα)-negative tumours in the MMTV-PyMT mouse mammary gland carcinoma model [59]. These CAF subsets secrete miR-22 via EVs to directly suppress the expression of ERα and PTEN in ERα-positive breast cancer cells and organoids, resulting in the induction of tamoxifen resistance. Interestingly, TIMP1, a CD63 ligand, can induce the STAT3 signalling pathway to regulate the expression of miR-22 and CD63 to generate a CD63-positive CAF phenotype. TIMP1 is upregulated in CD63-positive CAFs, suggesting a TIMP1-mediated positive feedback loop in the CD63-positive CAF phenotype. In addition, they showed that SFRS1 mediates miR-22 loading into CD63-positive CAF-derived EVs [59]. Although it is still unclear whether EV components differ among all CAF subsets, these findings indicate that CAF heterogeneity affects EV function in cancer progression.

Cancer-derived EVs reprogram energy metabolism in CAFs, as discussed in the previous section. CAFs also support the metabolic properties of cancer cells [60, 61]. Zhao et al. showed that CAF-derived EVs contain intact metabolites, such as amino acids, and change the metabolic properties of prostate cancer cells [60, 61]. Oxidative phosphorylation is inhibited in the presence of CAF-derived EVs. In contrast, glycolysis and lactate levels in prostate cancer cell lines are increased by these CAF-derived EVs. In addition to the direct transfer of metabolites via EVs, Li et al. also showed that the lncRNA SNHG3 affects the metabolic reprogramming of breast cancer cells. SNHG3 in EVs acts as a molecular sponge of miR-330-5p, resulting in increased lactate production in breast cancer cells [62]. Breast cancer-derived EVs transfer miR-122 and modify glucose consumption by fibroblasts at metastatic sites by targeting pyruvate kinases [63]. Moreover, metabolic dysregulation of CAFs may alter immunoregulation through IL-6 production [36]. Collectively, these findings imply that communication networks involving EVs contribute to cancer survival in environments with limited oxygen and nutrient supplies.

## Perspectives on the therapeutic and diagnostic application of CAF-derived EVs

As described above, EV-mediated interactions between cancer cells and CAFs have been implicated in several malignant behaviours of cancer cells. Therefore, targeting EVs and CAFs is being explored as a fascinating strategy for cancer therapy. Kong et al. suggested that the inhibition of vitamin D receptor (VDR) signalling could target miR-10 secretion via EVs from CAFs and inhibit its cancer-promoting functions in PDAC [64]. This study was motivated by the two reports demonstrating that VDR signalling reprograms CAFs, thus diminishing their cancer-promoting role in PDAC, and is associated with better clinical outcomes in CRC [65, 66]. The inhibitory effects of two compounds on the interplay between cancer cells and CAFs have also been demonstrated. Lee et al. showed the impact of the multitarget small drug HNC0014 on the function of cancer spheroid-derived EVs. HNC0014 inhibits CAF generation by these EVs and may contribute to the antitumour immune response [67]. Chen et al. utilized ovatodiolide (OV), the bioactive component of *Anisomeles indica*, to suppress CAF generation and tumour sphere formation induced by cancer-derived EVs [68]. Interestingly, proton-pump inhibitors (PPIs), which are used to eradicate the oncogenic pathogen *Helicobacter pylori*, may also inhibit CAF generation [69].

Some studies have identified the molecules specific to CAF-derived EVs and these molecules have been proposed as diagnostic biomarkers for cancer. Ganig et al. performed proteome analysis of EVs derived from CAFs and normal fibroblasts and identified 11 proteins with differential abundance in CAF-derived EVs [70]. Among these candidate proteins, QSOX1 was selected for further study and was found to be decreased in CAF-derived EVs compared with normal fibroblast-derived EVs. The abundance of QSOX1 in plasma samples was reduced in CRC patients compared to healthy donors and patients with benign disease [70]. Although this study used small cohorts to investigate QSOX1 in EVs, the findings suggest the utility of CAF-derived EVs in cancer diagnosis. The secretome of fibroblasts influenced by cancer-derived EVs has also been investigated as a biomarker for lung cancer detection. Zhang et al. compared the secretome profile of lung cancer-derived EV treated and untreated MCR5 fibroblasts to find candidates for a diagnostic marker [71]. Based on the multiple datasets comprising 1897 patient samples, they showed that the gene expression signatures of five candidates are independent prognostic factors for identifying patients who may require adjuvant therapy.

There remain challenges in the clinical application of EVs because of their diversity and the difficulty in selectively targeting cancer-associated EVs [8]. However, further investigations into targeting intercellular communication via EVs will provide several avenues for cancer therapy and diagnosis.

## Conclusion

The precise mechanisms of intercellular communication between cancer cells and CAFs remain obscure because diverse pathways modulate multiple EV components and non-EV factors, such as growth factors, cytokines and chemokines. In addition, CAFs are surprisingly heterogeneous, which is the major obstacle to understanding their biology and developing related therapeutics. However, as described above, remarkable progress in EV research has led to the elucidation of the novel mechanism underlying the intrinsic cancer cell–stromal cell interplay during cancer initiation and progression. EVs derived from cancer cells and CAFs have impressively diverse functions that contribute to tumour progression. Cancer cells secrete EVs and thus dictate specific properties in CAFs. Against such “education” by cancer cells, CAFs “respond” via EVs to suppress cancer initiation and progression. Understanding the precise mechanisms of EVs in cancer cell–CAF interactions may provide breakthroughs in the development of diagnostic and prognostic tools and therapeutic strategies for cancer.

## Data Availability

Not applicable.

## Abbreviations

CAFs: Cancer-associated fibroblasts
EVs: Extracellular vesicles
miRNAs: MicroRNAs
TME: Tumour microenvironment
MVBs: Multivesicular bodies
ESCRT: Endosomal sorting complex required for transport
BM-MSCs: Bone marrow-derived mesenchymal stem cells
α-SMA: Alpha-smooth muscle actin
EMT: Epithelial–mesenchymal transition
TGF-β: Transforming growth factor-beta
CXCL12: CXC-chemokine ligand 12
LIF: Leukaemia inhibitory factor
apCAFs: Antigen-presenting CAFs
ECM: Extracellular matrix
FGF2: Fibroblast growth factor 2
ITGB4: Integrin beta 4
BNIP3L: BCL2 interacting protein 3 like
B4GALT3: β-1,4-Galactosyltransferase III
lncRNAs: Long non-coding RNAs
PDAC: Pancreatic ductal adenocarcinoma
iCAFs: Inflammatory CAFs
myCAFs: Myofibroblastic CAFs
GC: Gastric cancer
Hmgb1: High-mobility group box 1
Tslp: Thymic stromal lymphopoietin
Irf1: Interferon regulatory factor 1
PCP: Planar cell polarity
DAMP: Damage-associated molecular pattern
ENT2: Equilibrative nucleoside transporter 2
CRC: Colorectal cancer
HuR: Human antigen R
ERα: Oestrogen receptor α
VDR: Vitamin D receptor

## References

[1] Joyce JA, Pollard JW (2009). Microenvironmental regulation of metastasis. Nat Rev Cancer.

[2] Sahai E, Astsaturov I, Cukierman E, DeNardo DG, Egeblad M, Evans RM, Fearon D, Greten FR, Hingorani SR, Hunter T (2020). A framework for advancing our understanding of cancer-associated fibroblasts. Nat Rev Cancer.

[3] Belli C, Trapani D, Viale G, D'Amico P, Duso BA, Della Vigna P, Orsi F, Curigliano G (2018). Targeting the microenvironment in solid tumors. Cancer Treat Rev.

[4] Kogure A, Yoshioka Y, Ochiya T (2020). Extracellular vesicles in cancer metastasis: potential as therapeutic targets and materials. Int J Mol Sci.

[5] Valadi H, Ekström K, Bossios A, Sjöstrand M, Lee JJ, Lötvall JO (2007). Exosome-mediated transfer of mRNAs and microRNAs is a novel mechanism of genetic exchange between cells. Nat Cell Biol.

[6] Yokoi A, Ochiya T (2021). Exosomes and extracellular vesicles: rethinking the essential values in cancer biology. Semin Cancer Biol.

[7] Hoshino A, Costa-Silva B, Shen TL, Rodrigues G, Hashimoto A, Tesic Mark M, Molina H, Kohsaka S, Di Giannatale A, Ceder S (2015). Tumour exosome integrins determine organotropic metastasis. Nature.

[8] Urabe F, Kosaka N, Ito K, Kimura T, Egawa S, Ochiya T (2020). Extracellular vesicles as biomarkers and therapeutic targets for cancer. Am J Physiol Cell Physiol.

[9] Théry C, Witwer KW, Aikawa E, Alcaraz MJ, Anderson JD, Andriantsitohaina R, Antoniou A, Arab T, Archer F, Atkin-Smith GK (2018). Minimal information for studies of extracellular vesicles 2018 (MISEV2018): a position statement of the International Society for Extracellular Vesicles and update of the MISEV2014 guidelines. J Extracell Vesicles.

[10] Lötvall J, Hill AF, Hochberg F, Buzás EI, Di Vizio D, Gardiner C, Gho YS, Kurochkin IV, Mathivanan S, Quesenberry P (2014). Minimal experimental requirements for definition of extracellular vesicles and their functions: a position statement from the International Society for Extracellular Vesicles. J Extracell Vesicles.

[11] Raposo G, Stoorvogel W (2013). Extracellular vesicles: exosomes, microvesicles, and friends. J Cell Biol.

[12] Muralidharan-Chari V, Clancy JW, Sedgwick A, D'Souza-Schorey C (2010). Microvesicles: mediators of extracellular communication during cancer progression. J Cell Sci.

[13] Akers JC, Gonda D, Kim R, Carter BS, Chen CC (2013). Biogenesis of extracellular vesicles (EV): exosomes, microvesicles, retrovirus-like vesicles, and apoptotic bodies. J Neurooncol.

[14] Elmore S (2007). Apoptosis: a review of programmed cell death. Toxicol Pathol.

[15] Kalluri R, Zeisberg M (2006). Fibroblasts in cancer. Nat Rev Cancer.

[16] Bartoschek M, Oskolkov N, Bocci M, Lövrot J, Larsson C, Sommarin M, Madsen CD, Lindgren D, Pekar G, Karlsson G (2018). Spatially and functionally distinct subclasses of breast cancer-associated fibroblasts revealed by single cell RNA sequencing. Nat Commun.

[17] Lambrechts D, Wauters E, Boeckx B, Aibar S, Nittner D, Burton O, Bassez A, Decaluwé H, Pircher A, Van den Eynde K (2018). Phenotype molding of stromal cells in the lung tumor microenvironment. Nat Med.

[18] Sugimoto H, Mundel TM, Kieran MW, Kalluri R (2006). Identification of fibroblast heterogeneity in the tumor microenvironment. Cancer Biol Ther.

[19] Raz Y, Cohen N, Shani O, Bell RE, Novitskiy SV, Abramovitz L, Levy C, Milyavsky M, Leider-Trejo L, Moses HL (2018). Bone marrow-derived fibroblasts are a functionally distinct stromal cell population in breast cancer. J Exp Med.

[20] Öhlund D, Handly-Santana A, Biffi G, Elyada E, Almeida AS, Ponz-Sarvise M, Corbo V, Oni TE, Hearn SA, Lee EJ (2017). Distinct populations of inflammatory fibroblasts and myofibroblasts in pancreatic cancer. J Exp Med.

[21] Biffi G, Oni TE, Spielman B, Hao Y, Elyada E, Park Y, Preall J, Tuveson DA (2019). IL1-induced JAK/STAT signaling is antagonized by TGFβ to shape CAF heterogeneity in pancreatic ductal adenocarcinoma. Cancer Discov.

[22] Massagué J (2008). TGFbeta in cancer. Cell.

[23] Orimo A, Gupta PB, Sgroi DC, Arenzana-Seisdedos F, Delaunay T, Naeem R, Carey VJ, Richardson AL, Weinberg RA (2005). Stromal fibroblasts present in invasive human breast carcinomas promote tumor growth and angiogenesis through elevated SDF-1/CXCL12 secretion. Cell.

[24] De Wever O, Nguyen QD, Van Hoorde L, Bracke M, Bruyneel E, Gespach C, Mareel M (2004). Tenascin-C and SF/HGF produced by myofibroblasts in vitro provide convergent pro-invasive signals to human colon cancer cells through RhoA and Rac. Faseb j.

[25] Su S, Chen J, Yao H, Liu J, Yu S, Lao L, Wang M, Luo M, Xing Y, Chen F (2018). CD10(+)GPR77(+) Cancer-associated fibroblasts promote cancer formation and chemoresistance by sustaining cancer stemness. Cell.

[26] Elyada E, Bolisetty M, Laise P, Flynn WF, Courtois ET, Burkhart RA, Teinor JA, Belleau P, Biffi G, Lucito MS (2019). Cross-species single-cell analysis of pancreatic ductal adenocarcinoma reveals antigen-presenting cancer-associated fibroblasts. Cancer Discov.

[27] Calvo F, Ege N, Grande-Garcia A, Hooper S, Jenkins RP, Chaudhry SI, Harrington K, Williamson P, Moeendarbary E, Charras G (2013). Mechanotransduction and YAP-dependent matrix remodelling is required for the generation and maintenance of cancer-associated fibroblasts. Nat Cell Biol.

[28] Gaggioli C, Hooper S, Hidalgo-Carcedo C, Grosse R, Marshall JF, Harrington K, Sahai E (2007). Fibroblast-led collective invasion of carcinoma cells with differing roles for RhoGTPases in leading and following cells. Nat Cell Biol.

[29] Labernadie A, Kato T, Brugués A, Serra-Picamal X, Derzsi S, Arwert E, Weston A, González-Tarragó V, Elosegui-Artola A, Albertazzi L (2017). A mechanically active heterotypic E-cadherin/N-cadherin adhesion enables fibroblasts to drive cancer cell invasion. Nat Cell Biol.

[30] Webber J, Steadman R, Mason MD, Tabi Z, Clayton A (2010). Cancer exosomes trigger fibroblast to myofibroblast differentiation. Cancer Res.

[31] Webber JP, Spary LK, Sanders AJ, Chowdhury R, Jiang WG, Steadman R, Wymant J, Jones AT, Kynaston H, Mason MD (2015). Differentiation of tumour-promoting stromal myofibroblasts by cancer exosomes. Oncogene.

[32] Huang Q, Hsueh CY, Shen YJ, Guo Y, Huang JM, Zhang YF, Li JY, Gong HL, Zhou L (2021). Small extracellular vesicle-packaged TGFβ1 promotes the reprogramming of normal fibroblasts into cancer-associated fibroblasts by regulating fibronectin in head and neck squamous cell carcinoma. Cancer Lett.

[33] Sung JS, Kang CW, Kang S, Jang Y, Chae YC, Kim BG, Cho NH (2020). ITGB4-mediated metabolic reprogramming of cancer-associated fibroblasts. Oncogene.

[34] Yan W, Wu X, Zhou W, Fong MY, Cao M, Liu J, Liu X, Chen CH, Fadare O, Pizzo DP (2018). Cancer-cell-secreted exosomal miR-105 promotes tumour growth through the MYC-dependent metabolic reprogramming of stromal cells. Nat Cell Biol.

[35] Sanford-Crane H, Abrego J, Sherman MH (2019). Fibroblasts as modulators of local and systemic cancer metabolism. Cancers (Basel).

[36] Valencia T, Kim JY, Abu-Baker S, Moscat-Pardos J, Ahn CS, Reina-Campos M, Duran A, Castilla EA, Metallo CM, Diaz-Meco MT (2014). Metabolic reprogramming of stromal fibroblasts through p62-mTORC1 signaling promotes inflammation and tumorigenesis. Cancer Cell.

[37] Martinez-Outschoorn UE, Lisanti MP, Sotgia F (2014). Catabolic cancer-associated fibroblasts transfer energy and biomass to anabolic cancer cells, fueling tumor growth. Semin Cancer Biol.

[38] Novo D, Heath N, Mitchell L, Caligiuri G, MacFarlane A, Reijmer D, Charlton L, Knight J, Calka M, McGhee E (2018). Mutant p53s generate pro-invasive niches by influencing exosome podocalyxin levels. Nat Commun.

[39] Ma S, McGuire MH, Mangala LS, Lee S, Stur E, Hu W, Bayraktar E, Villar-Prados A, Ivan C, Wu SY (2021). Gain-of-function p53 protein transferred via small extracellular vesicles promotes conversion of fibroblasts to a cancer-associated phenotype. Cell Rep.

[40] Tape CJ, Ling S, Dimitriadi M, McMahon KM, Worboys JD, Leong HS, Norrie IC, Miller CJ, Poulogiannis G, Lauffenburger DA (2016). Oncogenic KRAS regulates tumor cell signaling via stromal reciprocation. Cell.

[41] Vennin C, Mélénec P, Rouet R, Nobis M, Cazet AS, Murphy KJ, Herrmann D, Reed DA, Lucas MC, Warren SC (2019). CAF hierarchy driven by pancreatic cancer cell p53-status creates a pro-metastatic and chemoresistant environment via perlecan. Nat Commun.

[42] Hiebert P (2021). The Nrf2 transcription factor: a multifaceted regulator of the extracellular matrix. Matrix Biol Plus.

[43] Vu LT, Peng B, Zhang DX, Ma V, Mathey-Andrews CA, Lam CK, Kiomourtzis T, Jin J, McReynolds L, Huang L (2019). Tumor-secreted extracellular vesicles promote the activation of cancer-associated fibroblasts via the transfer of microRNA-125b. J Extracell Vesicles.

[44] Yoshii S, Hayashi Y, Iijima H, Inoue T, Kimura K, Sakatani A, Nagai K, Fujinaga T, Hiyama S, Kodama T (2019). Exosomal microRNAs derived from colon cancer cells promote tumor progression by suppressing fibroblast TP53 expression. Cancer Sci.

[45] Bhome R, Emaduddin M, James V, House LM, Thirdborough SM, Mellone M, Tulkens J, Primrose JN, Thomas GJ, De Wever O (2022). Epithelial to mesenchymal transition influences fibroblast phenotype in colorectal cancer by altering miR-200 levels in extracellular vesicles. J Extracell Vesicles.

[46] Naito Y, Yamamoto Y, Sakamoto N, Shimomura I, Kogure A, Kumazaki M, Yokoi A, Yashiro M, Kiyono T, Yanagihara K (2019). Cancer extracellular vesicles contribute to stromal heterogeneity by inducing chemokines in cancer-associated fibroblasts. Oncogene.

[47] Gener Lahav T, Adler O, Zait Y, Shani O, Amer M, Doron H, Abramovitz L, Yofe I, Cohen N, Erez N (2019). Melanoma-derived extracellular vesicles instigate proinflammatory signaling in the metastatic microenvironment. Int J Cancer.

[48] Goulet CR, Champagne A, Bernard G, Vandal D, Chabaud S, Pouliot F, Bolduc S (2019). Cancer-associated fibroblasts induce epithelial-mesenchymal transition of bladder cancer cells through paracrine IL-6 signalling. BMC Cancer.

[49] Fang T, Lv H, Lv G, Li T, Wang C, Han Q, Yu L, Su B, Guo L, Huang S (2018). Tumor-derived exosomal miR-1247-3p induces cancer-associated fibroblast activation to foster lung metastasis of liver cancer. Nat Commun.

[50] Luga V, Zhang L, Viloria-Petit AM, Ogunjimi AA, Inanlou MR, Chiu E, Buchanan M, Hosein AN, Basik M, Wrana JL (2012). Exosomes mediate stromal mobilization of autocrine Wnt-PCP signaling in breast cancer cell migration. Cell.

[51] Miki Y, Yashiro M, Okuno T, Kitayama K, Masuda G, Hirakawa K, Ohira M (2018). CD9-positive exosomes from cancer-associated fibroblasts stimulate the migration ability of scirrhous-type gastric cancer cells. Br J Cancer.

[52] Nigri J, Leca J, Tubiana SS, Finetti P, Guillaumond F, Martinez S, Lac S, Iovanna JL, Audebert S, Camoin L (2022). CD9 mediates the uptake of extracellular vesicles from cancer-associated fibroblasts that promote pancreatic cancer cell aggressiveness. Sci Signal.

[53] Boelens MC, Wu TJ, Nabet BY, Xu B, Qiu Y, Yoon T, Azzam DJ, Twyman-Saint Victor C, Wiemann BZ, Ishwaran H (2014). Exosome transfer from stromal to breast cancer cells regulates therapy resistance pathways. Cell.

[54] Nabet BY, Qiu Y, Shabason JE, Wu TJ, Yoon T, Kim BC, Benci JL, DeMichele AM, Tchou J, Marcotrigiano J (2017). Exosome RNA unshielding couples stromal activation to pattern recognition receptor signaling in cancer. Cell.

[55] Kunou S, Shimada K, Takai M, Sakamoto A, Aoki T, Hikita T, Kagaya Y, Iwamoto E, Sanada M, Shimada S (2021). Exosomes secreted from cancer-associated fibroblasts elicit anti-pyrimidine drug resistance through modulation of its transporter in malignant lymphoma. Oncogene.

[56] Deng X, Ruan H, Zhang X, Xu X, Zhu Y, Peng H, Zhang X, Kong F, Guan M (2020). Long noncoding RNA CCAL transferred from fibroblasts by exosomes promotes chemoresistance of colorectal cancer cells. Int J Cancer.

[57] Costa A, Kieffer Y, Scholer-Dahirel A, Pelon F, Bourachot B, Cardon M, Sirven P, Magagna I, Fuhrmann L, Bernard C (2018). Fibroblast heterogeneity and immunosuppressive environment in human breast cancer. Cancer Cell.

[58] Dou D, Ren X, Han M, Xu X, Ge X, Gu Y, Wang X (2026). Cancer-associated fibroblasts-derived exosomes suppress immune cell function in breast cancer via the miR-92/PD-L1 pathway. Front Immunol.

[59] Gao Y, Li X, Zeng C, Liu C, Hao Q, Li W, Zhang K, Zhang W, Wang S, Zhao H (2020). CD63(+) cancer-associated fibroblasts confer tamoxifen resistance to breast cancer cells through exosomal miR-22. Adv Sci (Weinh).

[60] Zhao H, Yang L, Baddour J, Achreja A, Bernard V, Moss T, Marini JC, Tudawe T, Seviour EG, San Lucas FA (2016). Tumor microenvironment derived exosomes pleiotropically modulate cancer cell metabolism. Elife.

[61] Achreja A, Zhao H, Yang L, Yun TH, Marini J, Nagrath D (2017). Exo-MFA–A 13C metabolic flux analysis framework to dissect tumor microenvironment-secreted exosome contributions towards cancer cell metabolism. Metab Eng.

[62] Li Y, Zhao Z, Liu W, Li X (2020). SNHG3 functions as miRNA sponge to promote breast cancer cells growth through the metabolic reprogramming. Appl Biochem Biotechnol.

[63] Fong MY, Zhou W, Liu L, Alontaga AY, Chandra M, Ashby J, Chow A, O'Connor ST, Li S, Chin AR (2015). Breast-cancer-secreted miR-122 reprograms glucose metabolism in premetastatic niche to promote metastasis. Nat Cell Biol.

[64] Kong F, Li L, Wang G, Deng X, Li Z, Kong X (2019). VDR signaling inhibits cancer-associated-fibroblasts' release of exosomal miR-10a-5p and limits their supportive effects on pancreatic cancer cells. Gut.

[65] Ferrer-Mayorga G, Gómez-López G, Barbáchano A, Fernández-Barral A, Peña C, Pisano DG, Cantero R, Rojo F, Muñoz A, Larriba MJ (2017). Vitamin D receptor expression and associated gene signature in tumour stromal fibroblasts predict clinical outcome in colorectal cancer. Gut.

[66] Sherman MH, Yu RT, Engle DD, Ding N, Atkins AR, Tiriac H, Collisson EA, Connor F, Van Dyke T, Kozlov S (2014). Vitamin D receptor-mediated stromal reprogramming suppresses pancreatitis and enhances pancreatic cancer therapy. Cell.

[67] Lee AH, Ghosh D, Quach N, Schroeder D, Dawson MR (2020). Ovarian cancer exosomes trigger differential biophysical response in tumor-derived fibroblasts. Sci Rep.

[68] Chen JH, Wu ATH, Bamodu OA, Yadav VK, Chao TY, Tzeng YM, Mukhopadhyay D, Hsiao M, Lee JC (2019). Ovatodiolide suppresses oral cancer malignancy by down-regulating exosomal Mir-21/STAT3/β-catenin cargo and preventing oncogenic transformation of normal gingival fibroblasts. Cancers (Basel).

[69] Guan XW, Zhao F, Wang JY, Wang HY, Ge SH, Wang X, Zhang L, Liu R, Ba Y, Li HL (2017). Tumor microenvironment interruption: a novel anti-cancer mechanism of Proton-pump inhibitor in gastric cancer by suppressing the release of microRNA-carrying exosomes. Am J Cancer Res.

[70] Ganig N, Baenke F, Thepkaysone ML, Lin K, Rao VS, Wong FC, Polster H, Schneider M, Helm D, Pecqueux M (2021). Proteomic analyses of fibroblast- and serum-derived exosomes identify QSOX1 as a marker for non-invasive detection of colorectal cancer. Cancers (Basel).

[71] Zhang J, Fu B, Li M, Mi S (2021). Secretome of activated fibroblasts induced by exosomes for the discovery of biomarkers in non-small cell lung cancer. Small.

[72] Ringuette Goulet C, Bernard G, Tremblay S, Chabaud S, Bolduc S, Pouliot F (2018). Exosomes induce fibroblast differentiation into cancer-associated fibroblasts through TGFβ signaling. Mol Cancer Res.

[73] El Buri A, Adams DR, Smith D, Tate RJ, Mullin M, Pyne S, Pyne NJ (2018). The sphingosine 1-phosphate receptor 2 is shed in exosomes from breast cancer cells and is N-terminally processed to a short constitutively active form that promotes extracellular signal regulated kinase activation and DNA synthesis in fibroblasts. Oncotarget.

[74] Li K, Liu T, Chen J, Ni H, Li W (2020). Survivin in breast cancer-derived exosomes activates fibroblasts by up-regulating SOD1, whose feedback promotes cancer proliferation and metastasis. J Biol Chem.

[75] Liang LJ, Yang Y, Wei WF, Wu XG, Yan RM, Zhou CF, Chen XJ, Wu S, Wang W, Fan LS (2021). Tumor-secreted exosomal Wnt2B activates fibroblasts to promote cervical cancer progression. Oncogenesis.

[76] Wu X, Zhou Z, Xu S, Liao C, Chen X, Li B, Peng J, Li D, Yang L (2020). Extracellular vesicle packaged LMP1-activated fibroblasts promote tumor progression via autophagy and stroma-tumor metabolism coupling. Cancer Lett.

[77] Gu J, Li X, Zhao L, Yang Y, Xue C, Gao Y, Li J, Han Q, Sun Z, Bai C (2021). The role of PKM2 nuclear translocation in the constant activation of the NF-κB signaling pathway in cancer-associated fibroblasts. Cell Death Dis.

[78] Wang D, Zhao C, Xu F, Zhang A, Jin M, Zhang K, Liu L, Hua Q, Zhao J, Liu J (2021). Cisplatin-resistant NSCLC cells induced by hypoxia transmit resistance to sensitive cells through exosomal PKM2. Theranostics.

[79] Huang J, Ding Z, Luo Q, Xu W (2019). Cancer cell-derived exosomes promote cell proliferation and inhibit cell apoptosis of both normal lung fibroblasts and non-small cell lung cancer cell through delivering alpha-smooth muscle actin. Am J Transl Res.

[80] Wei M, Yang T, Chen X, Wu Y, Deng X, He W, Yang J, Wang Z (2017). Malignant ascites-derived exosomes promote proliferation and induce carcinoma-associated fibroblasts transition in peritoneal mesothelial cells. Oncotarget.

[81] Zhang Y, Liu Z, Yang X, Lu W, Chen Y, Lin Y, Wang J, Lin S, Yun JP (2021). H3K27 acetylation activated-COL6A1 promotes osteosarcoma lung metastasis by repressing STAT1 and activating pulmonary cancer-associated fibroblasts. Theranostics.

[82] Novizio N, Belvedere R, Pessolano E, Tosco A, Porta A, Perretti M, Campiglia P, Filippelli A, Petrella A (2020). Annexin A1 released in extracellular vesicles by pancreatic cancer cells activates components of the tumor microenvironment, through interaction with the formyl-peptide receptors. Cells.

[83] Zhang YF, Zhou YZ, Zhang B, Huang SF, Li PP, He XM, Cao GD, Kang MX, Dong X, Wu YL (2019). Pancreatic cancer-derived exosomes promoted pancreatic stellate cells recruitment by pancreatic cancer. J Cancer.

[84] McAtee CO, Booth C, Elowsky C, Zhao L, Payne J, Fangman T, Caplan S, Henry MD, Simpson MA (2019). Prostate tumor cell exosomes containing hyaluronidase Hyal1 stimulate prostate stromal cell motility by engagement of FAK-mediated integrin signaling. Matrix Biol.

[85] Overmiller AM, Pierluissi JA, Wermuth PJ, Sauma S, Martinez-Outschoorn U, Tuluc M, Luginbuhl A, Curry J, Harshyne LA, Wahl JK (2017). Desmoglein 2 modulates extracellular vesicle release from squamous cell carcinoma keratinocytes. Faseb J.

[86] Ren Z, Lv M, Yu Q, Bao J, Lou K, Li X (2021). MicroRNA-370-3p shuttled by breast cancer cell-derived extracellular vesicles induces fibroblast activation through the CYLD/Nf-κB axis to promote breast cancer progression. Faseb J.

[87] Qin X, Lu M, Li G, Zhou Y, Liu Z (2021). Downregulation of tumor-derived exosomal miR-34c induces cancer-associated fibroblast activation to promote cholangiocarcinoma progress. Cancer Cell Int.

[88] Yang Y, Li J, Geng Y (2020). Exosomes derived from chronic lymphocytic leukaemia cells transfer miR-146a to induce the transition of mesenchymal stromal cells into cancer-associated fibroblasts. J Biochem.

[89] Wang J, Liu Y, Li Y, Zheng X, Gan J, Wan Z, Zhang J, Liu Y, Wang Y, Hu W (2021). Exosomal-miR-10a derived from colorectal cancer cells suppresses migration of human lung fibroblasts, and expression of IL-6, IL-8 and IL-1β. Mol Med Rep.

[90] Wang J, Guan X, Zhang Y, Ge S, Zhang L, Li H, Wang X, Liu R, Ning T, Deng T (2018). Exosomal miR-27a derived from gastric cancer cells regulates the transformation of fibroblasts into cancer-associated fibroblasts. Cell Physiol Biochem.

[91] Zhu G, Cao B, Liang X, Li L, Hao Y, Meng W, He C, Wang L, Li L (2021). Small extracellular vesicles containing miR-192/215 mediate hypoxia-induced cancer-associated fibroblast development in head and neck squamous cell carcinoma. Cancer Lett.

[92] Zhou Y, Ren H, Dai B, Li J, Shang L, Huang J, Shi X (2018). Hepatocellular carcinoma-derived exosomal miRNA-21 contributes to tumor progression by converting hepatocyte stellate cells to cancer-associated fibroblasts. J Exp Clin Cancer Res.

[93] Wei H, Wang J, Xu Z, Li W, Wu X, Zhuo C, Lu Y, Long X, Tang Q, Pu J (2021). Hepatoma cell-derived extracellular vesicles promote liver cancer metastasis by inducing the differentiation of bone marrow stem cells through microRNA-181d-5p and the FAK/Src pathway. Front Cell Dev Biol.

[94] Du C, Duan X, Yao X, Wan J, Cheng Y, Wang Y, Yan Y, Zhang L, Zhu L, Ni C (2020). Tumour-derived exosomal miR-3473b promotes lung tumour cell intrapulmonary colonization by activating the nuclear factor-κB of local fibroblasts. J Cell Mol Med.

[95] Fan J, Xu G, Chang Z, Zhu L, Yao J (2020). miR-210 transferred by lung cancer cell-derived exosomes may act as proangiogenic factor in cancer-associated fibroblasts by modulating JAK2/STAT3 pathway. Clin Sci (Lond).

[96] Lawson J, Dickman C, Towle R, Jabalee J, Javer A, Garnis C (2019). Extracellular vesicle secretion of miR-142-3p from lung adenocarcinoma cells induces tumor promoting changes in the stroma through cell-cell communication. Mol Carcinog.

[97] Hu T, Hu J (2019). Melanoma-derived exosomes induce reprogramming fibroblasts into cancer-associated fibroblasts via Gm26809 delivery. Cell Cycle.

[98] Shu S, Yang Y, Allen CL, Maguire O, Minderman H, Sen A, Ciesielski MJ, Collins KA, Bush PJ, Singh P (2018). Metabolic reprogramming of stromal fibroblasts by melanoma exosome microRNA favours a pre-metastatic microenvironment. Sci Rep.

[99] Zhou X, Yan T, Huang C, Xu Z, Wang L, Jiang E, Wang H, Chen Y, Liu K, Shao Z (2018). Melanoma cell-secreted exosomal miR-155-5p induce proangiogenic switch of cancer-associated fibroblasts via SOCS1/JAK2/STAT3 signaling pathway. J Exp Clin Cancer Res.

[100] Fan K, Spassova I, Gravemeyer J, Ritter C, Horny K, Lange A, Gambichler T, Ødum N, Schrama D, Schadendorf D (2021). Merkel cell carcinoma-derived exosome-shuttle miR-375 induces fibroblast polarization by inhibition of RBPJ and p53. Oncogene.

[101] Wang C, Wang Y, Chang X, Ba X, Hu N, Liu Q, Fang L, Wang Z (2020). Melanoma-derived exosomes endow fibroblasts with an invasive potential via miR-21 target signaling pathway. Cancer Manag Res.

[102] Ding L, Ren J, Zhang D, Li Y, Huang X, Hu Q, Wang H, Song Y, Ni Y, Hou Y (2018). A novel stromal lncRNA signature reprograms fibroblasts to promote the growth of oral squamous cell carcinoma via LncRNA-CAF/interleukin-33. Carcinogenesis.

[103] Cui Y, Wang D, Xie M (2021). Tumor-derived extracellular vesicles promote activation of carcinoma-associated fibroblasts and facilitate invasion and metastasis of ovarian cancer by carrying miR-630. Front Cell Dev Biol.

[104] Pang W, Su J, Wang Y, Feng H, Dai X, Yuan Y, Chen X, Yao W (2015). Pancreatic cancer-secreted miR-155 implicates in the conversion from normal fibroblasts to cancer-associated fibroblasts. Cancer Sci.

[105] Ghayad SE, Rammal G, Ghamloush F, Basma H, Nasr R, Diab-Assaf M, Chelala C, Saab R (2016). Exosomes derived from embryonal and alveolar rhabdomyosarcoma carry differential miRNA cargo and promote invasion of recipient fibroblasts. Sci Rep.

[106] Clerici SP, Peppelenbosch M, Fuhler G, Consonni SR, Ferreira-Halder CV (2021). Colorectal cancer cell-derived small extracellular vesicles educate human fibroblasts to stimulate migratory capacity. Front Cell Dev Biol.

[107] Rai A, Greening DW, Xu R, Suwakulsiri W, Simpson RJ (2020). Exosomes derived from the human primary colorectal cancer cell line SW480 orchestrate fibroblast-led cancer invasion. Proteomics.

[108] Huang YJ, Huang TH, Yadav VK, Sumitra MR, Tzeng DT, Wei PL, Shih JW, Wu AT (2020). Preclinical investigation of ovatodiolide as a potential inhibitor of colon cancer stem cells via downregulating sphere-derived exosomal β-catenin/STAT3/miR-1246 cargoes. Am J Cancer Res.

[109] Zhang D, Li D, Shen L, Hu D, Tang B, Guo W, Wang Z, Zhang Z, Wei G, He D (2020). Exosomes derived from Piwil2-induced cancer stem cells transform fibroblasts into cancer-associated fibroblasts. Oncol Rep.

[110] Ning X, Zhang H, Wang C, Song X (2018). Exosomes released by gastric cancer cells induce transition of pericytes into cancer-associated fibroblasts. Med Sci Monit.

[111] Gu J, Qian H, Shen L, Zhang X, Zhu W, Huang L, Yan Y, Mao F, Zhao C, Shi Y (2012). Gastric cancer exosomes trigger differentiation of umbilical cord derived mesenchymal stem cells to carcinoma-associated fibroblasts through TGF-β/Smad pathway. PLoS ONE.

[112] Goliwas KF, Ashraf HM, Wood AM, Wang Y, Hough KP, Bodduluri S, Athar M, Berry JL, Ponnazhagan S, Thannickal VJ (2021). Extracellular vesicle mediated tumor-stromal crosstalk within an engineered lung cancer model. Front Oncol.

[113] Di Vizio D, Morello M, Dudley AC, Schow PW, Adam RM, Morley S, Mulholland D, Rotinen M, Hager MH, Insabato L (2012). Large oncosomes in human prostate cancer tissues and in the circulation of mice with metastatic disease. Am J Pathol.

[114] Xu Z, Zheng X, Zheng J (2019). Tumor-derived exosomes educate fibroblasts to promote salivary adenoid cystic carcinoma metastasis via NGF-NTRK1 pathway. Oncol Lett.

[115] Shimoda M, Principe S, Jackson HW, Luga V, Fang H, Molyneux SD, Shao YW, Aiken A, Waterhouse PD, Karamboulas C (2014). Loss of the Timp gene family is sufficient for the acquisition of the CAF-like cell state. Nat Cell Biol.

[116] Chen Y, Zeng C, Zhan Y, Wang H, Jiang X, Li W (2017). Aberrant low expression of p85α in stromal fibroblasts promotes breast cancer cell metastasis through exosome-mediated paracrine Wnt10b. Oncogene.

[117] Hu Y, Yan C, Mu L, Huang K, Li X, Tao D, Wu Y, Qin J (2015). Fibroblast-derived exosomes contribute to chemoresistance through priming cancer stem cells in colorectal cancer. PLoS ONE.

[118] Oszvald Á, Szvicsek Z, Pápai M, Kelemen A, Varga Z, Tölgyes T, Dede K, Bursics A, Buzás EI, Wiener Z (2020). Fibroblast-derived extracellular vesicles induce colorectal cancer progression by transmitting amphiregulin. Front Cell Dev Biol.

[119] Zhao G, Li H, Guo Q, Zhou A, Wang X, Li P, Zhang S (2020). Exosomal Sonic Hedgehog derived from cancer-associated fibroblasts promotes proliferation and migration of esophageal squamous cell carcinoma. Cancer Med.

[120] Uchihara T, Miyake K, Yonemura A, Komohara Y, Itoyama R, Koiwa M, Yasuda T, Arima K, Harada K, Eto K (2020). Extracellular vesicles from cancer-associated fibroblasts containing annexin A6 induces FAK-YAP activation by stabilizing β1 integrin, enhancing drug resistance. Cancer Res.

[121] Grunberg N, Pevsner-Fischer M, Goshen-Lago T, Diment J, Stein Y, Lavon H, Mayer S, Levi-Galibov O, Friedman G, Ofir-Birin Y (2021). Cancer-associated fibroblasts promote aggressive gastric cancer phenotypes via heat shock factor 1-mediated secretion of extracellular vesicles. Cancer Res.

[122] Li W, Zhang X, Wang J, Li M, Cao C, Tan J, Ma D, Gao Q (2017). TGFβ1 in fibroblasts-derived exosomes promotes epithelial-mesenchymal transition of ovarian cancer cells. Oncotarget.

[123] Toti A, Santi A, Pardella E, Nesi I, Tomasini R, Mello T, Paoli P, Caselli A, Cirri P (2021). Activated fibroblasts enhance cancer cell migration by microvesicles-mediated transfer of Galectin-1. J Cell Commun Signal.

[124] You J, Li M, Cao LM, Gu QH, Deng PB, Tan Y, Hu CP (2019). Snail1-dependent cancer-associated fibroblasts induce epithelial-mesenchymal transition in lung cancer cells via exosomes. QJM.

[125] Zhang Y, Luo G, You S, Zhang L, Liang C, Chen X (2021). Exosomal LINC00355 derived from cancer-associated fibroblasts promotes bladder cancer cell proliferation and invasion by regulating miR-15a-5p/HMGA2 axis. Acta Biochim Biophys Sin (Shanghai).

[126] Yan L, Wang P, Fang W, Liang C (2020). Cancer-associated fibroblasts-derived exosomes-mediated transfer of LINC00355 regulates bladder cancer cell proliferation and invasion. Cell Biochem Funct.

[127] Chen B, Sang Y, Song X, Zhang D, Wang L, Zhao W, Liang Y, Zhang N, Yang Q (2021). Exosomal miR-500a-5p derived from cancer-associated fibroblasts promotes breast cancer cell proliferation and metastasis through targeting USP28. Theranostics.

[128] Liu Y, Yang Y, Du J, Lin D, Li F (2020). MiR-3613-3p from carcinoma-associated fibroblasts exosomes promoted breast cancer cell proliferation and metastasis by regulating SOCS2 expression. IUBMB Life.

[129] Wu HJ, Hao M, Yeo SK, Guan JL (2020). FAK signaling in cancer-associated fibroblasts promotes breast cancer cell migration and metastasis by exosomal miRNAs-mediated intercellular communication. Oncogene.

[130] Wang H, Wei H, Wang J, Li L, Chen A, Li Z (2020). MicroRNA-181d-5p-containing exosomes derived from CAFs promote EMT by regulating CDX2/HOXA5 in breast cancer. Mol Ther Nucleic Acids.

[131] Kim JE, Kim BG, Jang Y, Kang S, Lee JH, Cho NH (2020). The stromal loss of miR-4516 promotes the FOSL1-dependent proliferation and malignancy of triple negative breast cancer. Cancer Lett.

[132] Liu Y, Hua F, Zhan Y, Yang Y, Xie J, Cheng Y, Li F (2021). Carcinoma associated fibroblasts small extracellular vesicles with low miR-7641 promotes breast cancer stemness and glycolysis by HIF-1α. Cell Death Discov.

[133] Tao S, Li H, Ma X, Ma Y, He J, Gao Y, Li J (2021). Elevating microRNA-1-3p shuttled by cancer-associated fibroblasts-derived extracellular vesicles suppresses breast cancer progression and metastasis by inhibiting GLIS1. Cancer Gene Ther.

[134] Zhang Y, Wang S, Lai Q, Fang Y, Wu C, Liu Y, Li Q, Wang X, Gu C, Chen J (2020). Cancer-associated fibroblasts-derived exosomal miR-17-5p promotes colorectal cancer aggressive phenotype by initiating a RUNX3/MYC/TGF-β1 positive feedback loop. Cancer Lett.

[135] Chen X, Liu J, Zhang Q, Liu B, Cheng Y, Zhang Y, Sun Y, Ge H, Liu Y (2020). Exosome-mediated transfer of miR-93-5p from cancer-associated fibroblasts confer radioresistance in colorectal cancer cells by downregulating FOXA1 and upregulating TGFB3. J Exp Clin Cancer Res.

[136] Hu JL, Wang W, Lan XL, Zeng ZC, Liang YS, Yan YR, Song FY, Wang FF, Zhu XH, Liao WJ (2019). CAFs secreted exosomes promote metastasis and chemotherapy resistance by enhancing cell stemness and epithelial-mesenchymal transition in colorectal cancer. Mol Cancer.

[137] Ren J, Ding L, Zhang D, Shi G, Xu Q, Shen S, Wang Y, Wang T, Hou Y (2018). Carcinoma-associated fibroblasts promote the stemness and chemoresistance of colorectal cancer by transferring exosomal lncRNA H19. Theranostics.

[138] Bhome R, Goh RW, Bullock MD, Pillar N, Thirdborough SM, Mellone M, Mirnezami R, Galea D, Veselkov K, Gu Q (2017). Exosomal microRNAs derived from colorectal cancer-associated fibroblasts: role in driving cancer progression. Aging (Albany NY).

[139] Zhang HW, Shi Y, Liu JB, Wang HM, Wang PY, Wu ZJ, Li L, Gu LP, Cao PS, Wang GR (2021). Cancer-associated fibroblast-derived exosomal microRNA-24-3p enhances colon cancer cell resistance to MTX by down-regulating CDX2/HEPH axis. J Cell Mol Med.

[140] Chen X, Liu Y, Zhang Q, Liu B, Cheng Y, Zhang Y, Sun Y, Liu J (2021). Exosomal miR-590-3p derived from cancer-associated fibroblasts confers radioresistance in colorectal cancer. Mol Ther Nucleic Acids.

[141] Zheng Y, Zeng J, Lin D, Xia H, Wang X, Chen L, Chen H, Huang L, Zeng C (2021). Extracellular vesicles derived from cancer-associated fibroblast carries miR-224-5p targeting SLC4A4 to promote the proliferation, invasion and migration of colorectal cancer cells. Carcinogenesis.

[142] Zhou L, Li J, Tang Y, Yang M (2021). Exosomal LncRNA LINC00659 transferred from cancer-associated fibroblasts promotes colorectal cancer cell progression via miR-342-3p/ANXA2 axis. J Transl Med.

[143] Zhang N, Wang Y, Liu H, Shen W (2020). Extracellular vesicle encapsulated microRNA-320a inhibits endometrial cancer by suppression of the HIF1α/VEGFA axis. Exp Cell Res.

[144] Li BL, Lu W, Qu JJ, Ye L, Du GQ, Wan XP (2019). Loss of exosomal miR-148b from cancer-associated fibroblasts promotes endometrial cancer cell invasion and cancer metastasis. J Cell Physiol.

[145] Fan JT, Zhou ZY, Luo YL, Luo Q, Chen SB, Zhao JC, Chen QR (2021). Exosomal lncRNA NEAT1 from cancer-associated fibroblasts facilitates endometrial cancer progression via miR-26a/b-5p-mediated STAT3/YKL-40 signaling pathway. Neoplasia.

[146] Zhao Q, Huang L, Qin G, Qiao Y, Ren F, Shen C, Wang S, Liu S, Lian J, Wang D (2021). Cancer-associated fibroblasts induce monocytic myeloid-derived suppressor cell generation via IL-6/exosomal miR-21-activated STAT3 signaling to promote cisplatin resistance in esophageal squamous cell carcinoma. Cancer Lett.

[147] Shi L, Wang Z, Geng X, Zhang Y, Xue Z (2020). Exosomal miRNA-34 from cancer-associated fibroblasts inhibits growth and invasion of gastric cancer cells in vitro and in vivo. Aging (Albany NY).

[148] Zhang H, Deng T, Liu R, Ning T, Yang H, Liu D, Zhang Q, Lin D, Ge S, Bai M (2020). CAF secreted miR-522 suppresses ferroptosis and promotes acquired chemo-resistance in gastric cancer. Mol Cancer.

[149] Xu G, Zhang B, Ye J, Cao S, Shi J, Zhao Y, Wang Y, Sang J, Yao Y, Guan W (2019). Exosomal miRNA-139 in cancer-associated fibroblasts inhibits gastric cancer progression by repressing MMP11 expression. Int J Biol Sci.

[150] Shi H, Huang S, Qin M, Xue X, Guo X, Jiang L, Hong H, Fang J, Gao L (2021). Exosomal circ_0088300 derived from cancer-associated fibroblasts acts as a miR-1305 sponge and promotes gastric carcinoma cell tumorigenesis. Front Cell Dev Biol.

[151] Wang X, Qin X, Yan M, Shi J, Xu Q, Li Z, Yang W, Zhang J, Chen W (2019). Loss of exosomal miR-3188 in cancer-associated fibroblasts contributes to HNC progression. J Exp Clin Cancer Res.

[152] Qin X, Guo H, Wang X, Zhu X, Yan M, Wang X, Xu Q, Shi J, Lu E, Chen W (2019). Exosomal miR-196a derived from cancer-associated fibroblasts confers cisplatin resistance in head and neck cancer through targeting CDKN1B and ING5. Genome Biol.

[153] Yugawa K, Yoshizumi T, Mano Y, Itoh S, Harada N, Ikegami T, Kohashi K, Oda Y, Mori M (2021). Cancer-associated fibroblasts promote hepatocellular carcinoma progression through downregulation of exosomal miR-150-3p. Eur J Surg Oncol.

[154] Liu X, Wang H, Yang M, Hou Y, Chen Y, Bie P (2020). Exosomal miR-29b from cancer-associated fibroblasts inhibits the migration and invasion of hepatocellular carcinoma cells. Transl Cancer Res.

[155] Zhang Z, Li X, Sun W, Yue S, Yang J, Li J, Ma B, Wang J, Yang X, Pu M (2017). Loss of exosomal miR-320a from cancer-associated fibroblasts contributes to HCC proliferation and metastasis. Cancer Lett.

[156] Yang F, Yan Y, Yang Y, Hong X, Wang M, Yang Z, Liu B, Ye L (2020). MiR-210 in exosomes derived from CAFs promotes non-small cell lung cancer migration and invasion through PTEN/PI3K/AKT pathway. Cell Signal.

[157] Guo L, Li B, Yang J, Shen J, Ji J, Miao M (2020). Fibroblast-derived exosomal microRNA-369 potentiates migration and invasion of lung squamous cell carcinoma cells via NF1-mediated MAPK signaling pathway. Int J Mol Med.

[158] Zhang T, Zhang P, Li HX (2021). CAFs-derived exosomal miRNA-130a confers cisplatin resistance of NSCLC cells through PUM2-dependent packaging. Int J Nanomedicine.

[159] Wang H, Huang H, Wang L, Liu Y, Wang M, Zhao S, Lu G, Kang X (2021). Cancer-associated fibroblasts secreted miR-103a-3p suppresses apoptosis and promotes cisplatin resistance in non-small cell lung cancer. Aging (Albany NY).

[160] Sun LP, Xu K, Cui J, Yuan DY, Zou B, Li J, Liu JL, Li KY, Meng Z, Zhang B (2019). Cancer-associated fibroblast-derived exosomal miR-382-5p promotes the migration and invasion of oral squamous cell carcinoma. Oncol Rep.

[161] Li YY, Tao YW, Gao S, Li P, Zheng JM, Zhang SE, Liang J, Zhang Y (2018). Cancer-associated fibroblasts contribute to oral cancer cells proliferation and metastasis via exosome-mediated paracrine miR-34a-5p. EBioMedicine.

[162] Wang JW, Wu XF, Gu XJ, Jiang XH (2019). Exosomal miR-1228 from cancer-associated fibroblasts promotes cell migration and invasion of osteosarcoma by directly targeting SCAI. Oncol Res.

[163] Au Yeung CL, Co NN, Tsuruga T, Yeung TL, Kwan SY, Leung CS, Li Y, Lu ES, Kwan K, Wong KK (2016). Exosomal transfer of stroma-derived miR21 confers paclitaxel resistance in ovarian cancer cells through targeting APAF1. Nat Commun.

[164] Guo H, Ha C, Dong H, Yang Z, Ma Y, Ding Y (2019). Cancer-associated fibroblast-derived exosomal microRNA-98-5p promotes cisplatin resistance in ovarian cancer by targeting CDKN1A. Cancer Cell Int.

[165] Fang Y, Zhou W, Rong Y, Kuang T, Xu X, Wu W, Wang D, Lou W (2019). Exosomal miRNA-106b from cancer-associated fibroblast promotes gemcitabine resistance in pancreatic cancer. Exp Cell Res.

[166] Josson S, Gururajan M, Sung SY, Hu P, Shao C, Zhau HE, Liu C, Lichterman J, Duan P, Li Q (2015). Stromal fibroblast-derived miR-409 promotes epithelial-to-mesenchymal transition and prostate tumorigenesis. Oncogene.

[167] Liu Y, Fu W, Cao X, Li S, Xiong T, Zhang X, Wu X, Cheng L, Wei Y, Gao B (2021). Delivery of miR-224-5p by exosomes from cancer-associated fibroblasts potentiates progression of clear cell renal cell carcinoma. Comput Math Methods Med.

[168] Sansone P, Savini C, Kurelac I, Chang Q, Amato LB, Strillacci A, Stepanova A, Iommarini L, Mastroleo C, Daly L (2017). Packaging and transfer of mitochondrial DNA via exosomes regulate escape from dormancy in hormonal therapy-resistant breast cancer. Proc Natl Acad Sci U S A.

[169] Dourado MR, Korvala J, Åström P, De Oliveira CE, Cervigne NK, Mofatto LS, Campanella Bastos D, Pereira Messetti AC, Graner E, Paes Leme AF (2019). Extracellular vesicles derived from cancer-associated fibroblasts induce the migration and invasion of oral squamous cell carcinoma. J Extracell Vesicles.

[170] Richards KE, Zeleniak AE, Fishel ML, Wu J, Littlepage LE, Hill R (2017). Cancer-associated fibroblast exosomes regulate survival and proliferation of pancreatic cancer cells. Oncogene.

[171] Scognamiglio I, Cocca L, Puoti I, Palma F, Ingenito F, Quintavalle C, Affinito A, Roscigno G, Nuzzo S, Chianese RV (2022). Exosomal microRNAs synergistically trigger stromal fibroblasts in breast cancer. Mol Ther Nucleic Acids.

[172] Feng T, Fang F, Zhang C, Li T, He J, Shen Y, Yu H, Liu X (2022). Fluid shear stress-induced exosomes from liver cancer cells promote activation of cancer-associated fibroblasts via IGF2-PI3K axis. Front Biosci (Landmark Ed).

[173] Tang H, Zhou X, Zhao X, Luo X, Luo T, Chen Y, Liang W, Jiang E, Liu K, Shao Z (2022). HSP90/IKK-rich small extracellular vesicles activate pro-angiogenic melanoma-associated fibroblasts via the NF-κB/CXCL1 axis. Cancer Sci.

[174] Wang B, Zhang S, Tong F, Wang Y, Wei L (2022). HPV(+) HNSCC-derived exosomal miR-9-5p inhibits TGF-β signaling-mediated fibroblast phenotypic transformation through NOX4. Cancer Sci.

[175] Fong MY, Yan W, Ghassemian M, Wu X, Zhou X, Cao M, Jiang L, Wang J, Liu X, Zhang J (2021). Cancer-secreted miRNAs regulate amino-acid-induced mTORC1 signaling and fibroblast protein synthesis. EMBO Rep.

[176] Zhang DX, Dang XTT, Vu LT, Lim CMH, Yeo EYM, Lam BWS, Leong SM, Omar N, Putti TC, Yeh YC (2022). αvβ1 integrin is enriched in extracellular vesicles of metastatic breast cancer cells: A mechanism mediated by galectin-3. J Extracell Vesicles.

[177] Lee PJ, Sui YH, Liu TT, Tsang NM, Huang CH, Lin TY, Chang KP, Liu SC (2022). Epstein-Barr viral product-containing exosomes promote fibrosis and nasopharyngeal carcinoma progression through activation of YAP1/FAPα signaling in fibroblasts. J Exp Clin Cancer Res.

[178] Rao VS, Gu Q, Tzschentke S, Lin K, Ganig N, Thepkaysone ML, Wong FC, Polster H, Seifert L, Seifert AM (2022). Extravesicular TIMP-1 is a non-invasive independent prognostic marker and potential therapeutic target in colorectal liver metastases. Oncogene.

[179] Ahn S, Kwon A, Huh YH, Rhee S, Song WK (2022). Tumor-derived miR-130b-3p induces cancer-associated fibroblast activation by targeting SPIN90 in luminal A breast cancer. Oncogenesis.

[180] Zhang C, Wang XY, Zhang P, He TC, Han JH, Zhang R, Lin J, Fan J, Lu L, Zhu WW (2022). Cancer-derived exosomal HSPC111 promotes colorectal cancer liver metastasis by reprogramming lipid metabolism in cancer-associated fibroblasts. Cell Death Dis.

[181] Wang D, Wang X, Song Y, Si M, Sun Y, Liu X, Cui S, Qu X, Yu X (2022). Exosomal miR-146a-5p and miR-155-5p promote CXCL12/CXCR7-induced metastasis of colorectal cancer by crosstalk with cancer-associated fibroblasts. Cell Death Dis.

[182] Inoue T, Hayashi Y, Tsujii Y, Yoshii S, Sakatani A, Kimura K, Uema R, Kato M, Saiki H, Shinzaki S (2021). Suppression of autophagy promotes fibroblast activation in p53-deficient colorectal cancer cells. Sci Rep.

[183] Jiang Y, Wang K, Lu X, Wang Y, Chen J (2021). Cancer-associated fibroblasts-derived exosomes promote lung cancer progression by OIP5-AS1/ miR-142-5p/ PD-L1 axis. Mol Immunol.

[184] Chen H, Zhao L, Meng Y, Qian X, Fan Y, Zhang Q, Wang C, Lin F, Chen B, Xu L (2022). Sulfonylurea receptor 1-expressing cancer cells induce cancer-associated fibroblasts to promote non-small cell lung cancer progression. Cancer Lett.

[185] Lu L, Huang J, Mo J, Da X, Li Q, Fan M, Lu H (2022). Exosomal lncRNA TUG1 from cancer-associated fibroblasts promotes liver cancer cell migration, invasion, and glycolysis by regulating the miR-524-5p/SIX1 axis. Cell Mol Biol Lett.

[186] Zhang Y, Zhao J, Ding M, Su Y, Cui D, Jiang C, Zhao S, Jia G, Wang X, Ruan Y (2020). Loss of exosomal miR-146a-5p from cancer-associated fibroblasts after androgen deprivation therapy contributes to prostate cancer metastasis. J Exp Clin Cancer Res.

[187] Tan D, Li G, Zhang P, Peng C, He B (2022). LncRNA SNHG12 in extracellular vesicles derived from carcinoma-associated fibroblasts promotes cisplatin resistance in non-small cell lung cancer cells. Bioengineered.

[188] Yang K, Zhang F, Luo B, Qu Z (2022). CAFs-derived small extracellular vesicles circN4BP2L2 promotes proliferation and metastasis of colorectal cancer via miR-664b-3p/HMGB3 pathway. Cancer Biol Ther.

[189] Dai X, Xie Y, Dong M (2022). Cancer-associated fibroblasts derived extracellular vesicles promote angiogenesis of colorectal adenocarcinoma cells through miR-135b-5p/FOXO1 axis. Cancer Biol Ther.

[190] Richards KE, Xiao W, Hill R, On Behalf Of The Usc Pancreas Research T (2022). Cancer-associated fibroblasts confer gemcitabine resistance to pancreatic cancer cells through PTEN-targeting miRNAs in exosomes. Cancers (Basel).

[191] Han Y, Qian X, Xu T, Shi Y (2022). Carcinoma-associated fibroblasts release microRNA-331-3p containing extracellular vesicles to exacerbate the development of pancreatic cancer via the SCARA5-FAK axis. Cancer Biol Ther.

[192] Shi L, Zhu W, Huang Y, Zhuo L, Wang S, Chen S, Zhang B, Ke B (2022). Cancer-associated fibroblast-derived exosomal microRNA-20a suppresses the PTEN/PI3K-AKT pathway to promote the progression and chemoresistance of non-small cell lung cancer. Clin Transl Med.

[193] Pan S, Deng Y, Fu J, Zhang Y, Zhang Z, Qin X (2022). N6-methyladenosine upregulates miR-181d-5p in exosomes derived from cancer-associated fibroblasts to inhibit 5-FU sensitivity by targeting NCALD in colorectal cancer. Int J Oncol.

[194] Sun L, Ke M, Wang X, Yin M, Wei J, Xu L, Tian X, Wang F, Zhang H, Fu S (2022). FAP(high) α-SMA(low) cancer-associated fibroblast-derived SLPI protein encapsulated in extracellular vesicles promotes ovarian cancer development via activation of PI3K/AKT and downstream signaling pathways. Mol Carcinog.

[195] Yan Z, Sheng Z, Zheng Y, Feng R, Xiao Q, Shi L, Li H, Yin C, Luo H, Hao C (2021). Cancer-associated fibroblast-derived exosomal miR-18b promotes breast cancer invasion and metastasis by regulating TCEAL7. Cell Death Dis.

[196] Zhao J, Lin H, Huang K, Li S (2022). Cancer-associated fibroblasts-derived extracellular vesicles carrying lncRNA SNHG3 facilitate colorectal cancer cell proliferation via the miR-34b-5p/HuR/HOXC6 axis. Cell Death Discov.

[197] Wang S, Du P, Cao Y, Ma J, Yang X, Yu Z, Yang Y (2022). Cancer associated fibroblasts secreted exosomal miR-1290 contributes to prostate cancer cell growth and metastasis via targeting GSK3β. Cell Death Discov.

[198] Fang F, Guo C, Zheng W, Wang Q, Zhou L (2022). Exosome-mediated transfer of miR-1323 from cancer-associated fibroblasts confers radioresistance of C33A cells by targeting PABPN1 and activating Wnt/β-catenin signaling pathway in cervical cancer. Reprod Sci.

[199] Shi Z, Jiang T, Cao B, Sun X, Liu J (2022). CAF-derived exosomes deliver LINC01410 to promote epithelial–mesenchymal transition of esophageal squamous cell carcinoma. Exp Cell Res.

[200] Qin W, Wang L, Tian H, Wu X, Xiao C, Pan Y, Fan M, Tai Y, Liu W, Zhang Q (2022). CAF-derived exosomes transmitted Gremlin-1 promotes cancer progression and decreases the sensitivity of hepatoma cells to sorafenib. Mol Carcinog.

[201] Wang Y, Li T, Yang L, Zhang X, Wang X, Su X, Ji C, Wang Z (2022). Cancer-associated fibroblast-released extracellular vesicles carrying miR-199a-5p induces the progression of​ gastric cancer through regulation of FKBP5-mediated AKT1/mTORC1 signaling pathway. Cell Cycle.

[202] Yin H, Yu S, Xie Y, Dai X, Dong M, Sheng C, Hu J (2021). Cancer-associated fibroblasts-derived exosomes upregulate microRNA-135b-5p to promote colorectal cancer cell growth and angiogenesis by inhibiting thioredoxin-interacting protein. Cell Signal.

